# Non-Invasive Measurement of Exercise-Induced Oxidative Stress in Response to Physical Activity. A Systematic Review and Meta-Analysis

**DOI:** 10.3390/antiox10122008

**Published:** 2021-12-17

**Authors:** Giulia Squillacioti, Fulvia Guglieri, Nicoletta Colombi, Federica Ghelli, Paola Berchialla, Paolo Gardois, Roberto Bono

**Affiliations:** 1 Department of Public Health and Pediatrics, University of Turin, 10126 Turin, Italy; fulvia.guglieri@unito.it (F.G.); federica.ghelli@unito.it (F.G.); roberto.bono@unito.it (R.B.); 2 Biblioteca Federata di Medicina Ferdinando Rossi, University of Turin, 10126 Turin, Italy; nicoletta.colombi@unito.it (N.C.); paolo.gardois@unito.it (P.G.); 3 Dipartimento di Scienze Cliniche e Biologiche, University of Turin, 10043 Turin, Italy; paola.berchialla@unito.it

**Keywords:** physical activity, public health, oxidative stress, non-invasive biomarker, saliva, urine

## Abstract

Physical activity may benefit health by modulating oxidative stress and inflammation. However, the selection of suitable exercise-induced oxidative stress biomarkers is still challenging. This study aimed at systematically summarizing the available evidence on exercise-induced oxidative stress measured in urine and/or saliva. Two meta-analyses including the most frequently quantified biomarkers of oxidative stress, namely, urinary isoprostane and DNA oxidation products, were performed. Three electronic databases (PubMed, EMBASE and Cochrane CENTRAL) were interrogated. Among 4479 records, 43 original articles were included in the systematic review and 11 articles were included in meta-analysis I and II, respectively. We observed a pooled trend of increase of urinary isoprostanes in response to physical activity (+0.95, 95% CI: −0.18; 2.09). In comparison with aerobic exercise, anaerobic training determined a greater induction of isoprostanes (+5.21, 95% CI: 2.76; 7.66, *p* < 0.0001), which were markedly increased after vigorous physical activity (+6.01, 95% CI: 1.18; 10.84, *p* < 0.001) and slightly decreased in response to exercise interventions protracted over time (e.g., months) (−1.19, 95% CI: −2.25; −0.12, *p* < 0.001). We recommend the most integrative approach of oxidative stress multi-marker panels in response to physical activity instead of selecting one preferential biomarker to quantify physical activity-induced oxidative stress in humans.

## 1. Introduction

In the last few decades, the scientific interest in physical activity-induced oxidative stress has been fuelled by three complementary concepts. First, physical activity helps prevent chronic diseases and improve health [1]. Second, oxidative stress is involved in the pathogenesis [2] or represents a downstream consequence of several diseases [3]. Third, physical activity influences cellular redox homeostasis and, thus, oxidative status in humans [4]. Since the pioneering discovery that lipid peroxidation biomarkers increase in subjects following acute exercise [5,6], the understanding of the exercise-induced oxidative stress was further extended by the introduction of some key scientific discoveries including (1) the involvement of pro-oxidants species in the production and modulation of muscles force [7]; (2) the dose-response effect of training on primary antioxidant levels in cardiac and skeletal muscle [8]; (3) the contribution of nitric oxide in muscle vasodilatation [9] and its production in contracting muscles [10]. Overall, this body of evidence laid the foundations for applying the theory of hormesis to exercise-induced oxidative stress [11]. In general terms, hormesis has been defined as a “process in which a low dose of a chemical agent or environmental factor that is damaging at high doses, induces an adaptive beneficial effect on the cells or organism” [12]. In this regard, acute and vigorous physical activity has been linked to increased reactive oxygen species (ROS) [13], while moderate and regular physical activity can enhance muscle oxidative capacity [14], muscle force production [15] and general antioxidant responses, acting as an adaptive stimulus against an overload of ROS [11,15,16]. Hence, based on the intensity [17] and duration [13], physical activity may act either as a natural antioxidant or as an oxidative stress trigger. Irrespective of their generation and source of production, ROS are highly reactive and have a short life; thus, they are commonly measured indirectly. In fact, under the attack of ROS, biological molecules undergo enzymatic and non-enzymatic reactions producing several by-products, which serve as biomarkers of oxidative stress [18]. Although several biomarkers of oxidative stress have been already validated in human populations [19], the quantification of exercise-induced oxidative stress biomarkers is still challenging, especially in large-scale studies where the cost-effectiveness and non-invasive nature of measures are crucial.

Therefore, the general aim of this systematic review and meta-analysis was to summarise the actual knowledge on biomarkers of oxidative stress collected in a non-invasive way in adults who performed physical activity. The specific aim was twofold: first, we sought to review both urinary and salivary biomarkers of oxidative stress induced by physical activity, and second, we aimed at disentangling the influence of physical activity on two extensively quantified oxidative stress biomarkers, namely, isoprostanes and DNA oxidation products. The present work is to support the applied research in free radical biology, physical activity-induced oxidative stress and health in humans.

## 2. Materials and Methods

This study is reported according to the recommendations from “Preferred Reporting Items for Systematic Reviews and Meta-Analyses” PRISMA [20] and was conducted according to the registered PROSPERO protocol (Protocol n. CRD42020188391). At the time of the first protocol submission, the PROSPERO platform was prioritising the registration of COVID-19 protocols. Therefore, to allow the PROSPERO team to focus on COVID-19 and to avoid further delay, the current submission passed a basic automated check and was published automatically.

### 2.1. Search Strategy

The bibliographic research was conducted on 28 April 2020. Three electronic databases, namely PubMed, EMBASE and Cochrane CENTRAL were interrogated. Keywords relying on “oxidative stress” AND “physical activity” AND “urine OR “saliva” were searched. Further details on the full search string are available in Appendix A.

### 2.2. Eligibility Criteria

Studies were included if they were: (i) original research; (ii) involving adult subjects (18+ years); (iii) reporting measurements of oxidative stress biomarkers in urine and/or saliva; (iv) including physical activity and/or exercise as the main independent variable; (v) reporting measurements of oxidative stress biomarkers both pre and post a physical activity/exercise intervention; (vi) published in English or Italian language.

Systematic reviews, scoping reviews, case studies, editorials, conference papers/abstracts and all primary research reporting non-quantitative data, based on animal or in vitro experiments, with an ecological design, were excluded. Moreover, studies involving any kind of antioxidant supplementation, non-objectively assessing physical activity (e.g., questionnaire), including physical activity performed under extreme conditions (e.g., high altitude) were excluded.

### 2.3. Data Extraction

Two independent reviewers used a spreadsheet specifically customised to cope with the data extraction process. If data were originally reported according to particular subgroups (e.g., sex, age classes, smoking habits, physical activity intensities), all data were extracted. Biomarker variations described throughout this systematic review are in reference to the resting condition (i.e., baseline). If biomarker measurements at different time points were available, only the first time point following physical activity was extracted. Data on urinary isoprostane were generally reported as “isoprostanes” because several articles did not specify if free, total or specific isomers of isoprostane were analysed. Data originally presented by graphs were extracted by the Web plot digitizer software (https://apps.automeris.io/wpd/).

### 2.4. Quality Assessment

The risk of bias (quality) assessment was appraised using three specific tools based on the study design of the included researches, namely: (1) the National Institute of Health (NIH) Quality Assessment Tools for observational, case-series, cross-sectional and before-after studies were used for a critical appraisal of the internal validity of the studies [21]; (2) the PEDro scale (available at: https://pedro.org.au/english/resources/pedro-scale/ Accessed on 1 July 2020) to appraise the randomised controlled trials; and (3) the Johanna Briggs Institute checklist [22] was used to check the methodological quality of the quasi-experimental trials (i.e., non-randomised). Since each tool adopts different ratings, we expressed our quality rating as a percentage and the quality score underwent re-coding based on the tertiles (1st tertile = poor quality; 2nd tertile = medium quality; 3rd tertile = high quality).

### 2.5. Statistical Methods

Continuous variables were summarised and reported as mean ± standard deviation (SD) or median ± inter quartile range (IQR) or mean ± standard error of the mean (SEM). Methods in [23] were used to approximate the SD from the sample size, median and IQR.

For each study, the mean change from baseline was computed. Since the correlation coefficient r between post score and pre score is needed for computing the standard error, the value r = 0.7 was imputed as suggested by Rosenthal [24]. Then, effect sizes were computed as standardised mean differences based on the Hedges’ g method.

To estimate the pooled effect of the physical activity intervention on oxidative stress biomarkers a random-effect meta-analytic model with the DerSimonian–Laird estimator (inverse variance method) was used. The average effect size and a 95% Confidence Interval (CI) were computed by the Jackson method. The heterogeneity among the studies was inspected by the Cochran’s Q test and the Higgins I^2^ statistics.

Publication bias was assessed by visual inspection of funnel plots and by carrying out the Eggers’ test. Meta-regression models were built to check the influence of (i) the quality of the included studies; (ii) types of physical activity, (iii) duration of physical activity (i.e., “acute” or “chronic”) and (iv) intensities of physical activity on the relationship between oxidative stress biomarker and physical activity. Results were expressed as regression coefficients (95% CI).

We performed a set of sensitivity analyses to: (i) identify influential studies that resulted in variation, using graphic display of heterogeneity plots, which fit the same meta-analysis model for all the possible study combinations and look for specific patterns performing clustering with k-means, DBSCAN and Gaussian mixed models [25]; (ii) to check for the outliers and the influence of each included article on the overall heterogeneity and (iii) to evaluate the studies that were more contributing to the heterogeneity, previously identified by the Baujat diagnostic and plot. Studies reporting spot urine biomarkers without any normalisations to creatinine and/or data referred to subgroups not relevant to the research question (e.g., smokers versus non-smokers) were excluded from the meta-analysis. All the analyses have been performed using R, version 4.0.2 [26]

## 3. Results

### 3.1. Qualitative Synthesis

**Figure 1:**
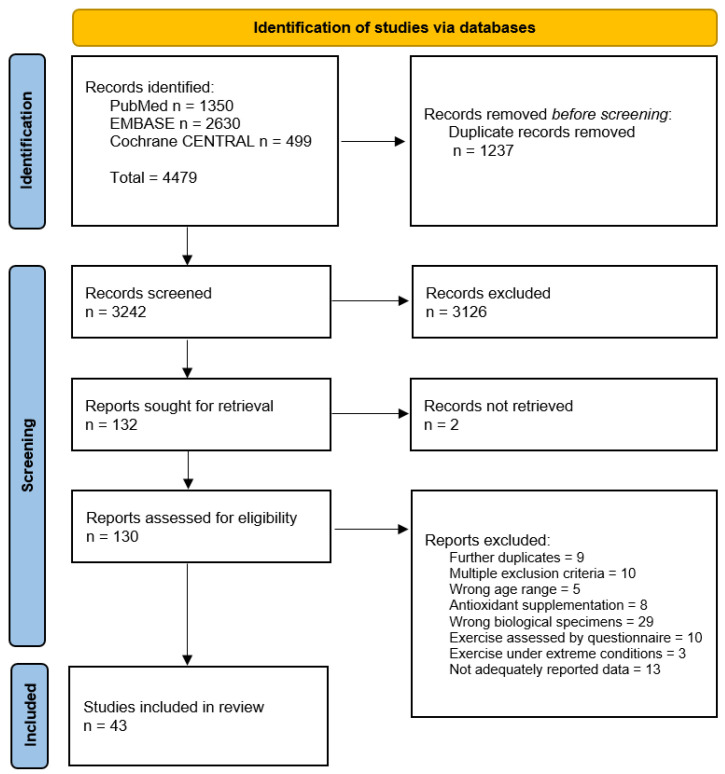
Step-by-step study selection process. Modified from: [20].

A total of 4479 studies were initially identified, 3242 full-text articles screened, 43 peer-reviewed studies included in the Systematic Review [27,28,29,30,31,32,33,34,35,36,37,38,39,40,41,42,43,44,45,46,47,48,49,50,51,52,53,54,55,56,57,58,59,60,61,62,63,64,65,66,67,68,69] and 11 articles included in the meta-analysis on DNA oxidation products [27,28,36,37,52,53,54,59,64,66,69] and on isoprostanes [32,39,40,41,42,44,49,57,59,62,63], respectively (Figure 1). The main reasons for exclusion relied on duplicates remaining after the deduplication step, not available full texts (e.g., conference paper), original data not adequately reported (e.g., missing), or not eligible studies for numerous reasons (e.g., age range, antioxidant supplementation, biomarkers measured in other specimens than saliva and urine).

#### 3.1.1. Study and Participant Characteristics

Table 1 presents a summary of the included studies. The majority of the studies (47%) were uncontrolled experiments employing a before-after design (*n* = 20), while a cumulated 49% consisted of controlled before-after, longitudinal and randomised controlled trials (*n* = 7, respectively). One study used randomised cross-over design and another was self-controlled case-series. Studies were mainly located in Japan (*n* = 4), Brazil (*n* = 4), Italy (*n* = 4), Spain (*n* = 4) and USA (*n* = 4) followed by Canada (*n* = 3), Netherlands (*n* = 3), Iran (*n* = 2) and Denmark, Egypt, Germany, Greece, India and Mexico (*n* = 1, respectively). Nine studies did not clearly state the study location. The studies were published from 1993 to 2019.

Sample sizes ranged from 5 to 98 subjects. Overall, 957 adults aged between 19 and 72 years (39.8 ± 18.2 years) were included in the systematic review and 70% of them were males (*n* = 671). A total of 60% of subjects were healthy, 10% were not classified, while a cumulated 30% reported a disease among diabetes or obesity (13%), respiratory diseases (9%) and a miscellaneous of other pathologies including arthritis rheumatoid, cancer and periodontitis (8%). Subjects were generally active (41%) or professional athletes (24%), whereas 33% of them reported a sedentary lifestyle and 2% did not provide any details. Only 27% of them performed low-intensity physical activity and 42% were engaged in high-intensity or medium-intensity (25%) physical activity protocols (for 6% of them the physical activity intensity has not been specified). A total of 5% of subjects underwent both aerobic and anaerobic exercises, while the remaining followed aerobic (67%) or anaerobic (27%) protocols.

#### 3.1.2. Oxidative Stress Biomarkers

Table 2 summarises key characteristics of biomarkers of oxidative stress. The two most investigated oxidative stress biomarkers were urinary isoprostanes and DNA oxidation products (i.e., 8-oxo-dG or 8-OH-dG). In particular, 44% of the studies focused on DNA oxidation products (*n* = 19), 40% analysed isoprostanes (*n* = 17) and the remaining 16% included a large variety of other biomarkers: (i) measured in saliva, such as peroxidase, lipid hydroperoxides, superoxide dismutase, catalase, total antioxidant status or capacity, advanced oxidation protein products, glutathione, vitamin C and Uric Acid (UA); (ii) measured in urine, such as allantoin, hydrogen peroxide and urate; (iii) measured in both urine and saliva such as malondialdehyde (MDA).

A total of 84% of the studies quantified oxidative stress biomarkers in urine, either using spot urine (*n* = 21), either 24 h (*n* = 11) or 12 h urine (*n* = 4), while eight articles out of 43 used saliva specimens (19%). The most widely performed analytical technique was ELISA (49% of the studies), followed by HPLC (26%), while the remaining 25% of the studies used radioimmunoassay (*n* = 2), GC-MS, tandem mass spectrometry and ultra-performance liquid chromatography and flow cytometry (*n* = 1, respectively) or other analytical techniques generally defined as “colorimetric”, “spectrophotometric” or “enzymatic”.

#### 3.1.3. The Effect of Physical Activity on Oxidative Stress Biomarkers in Saliva

**Figure 2:**
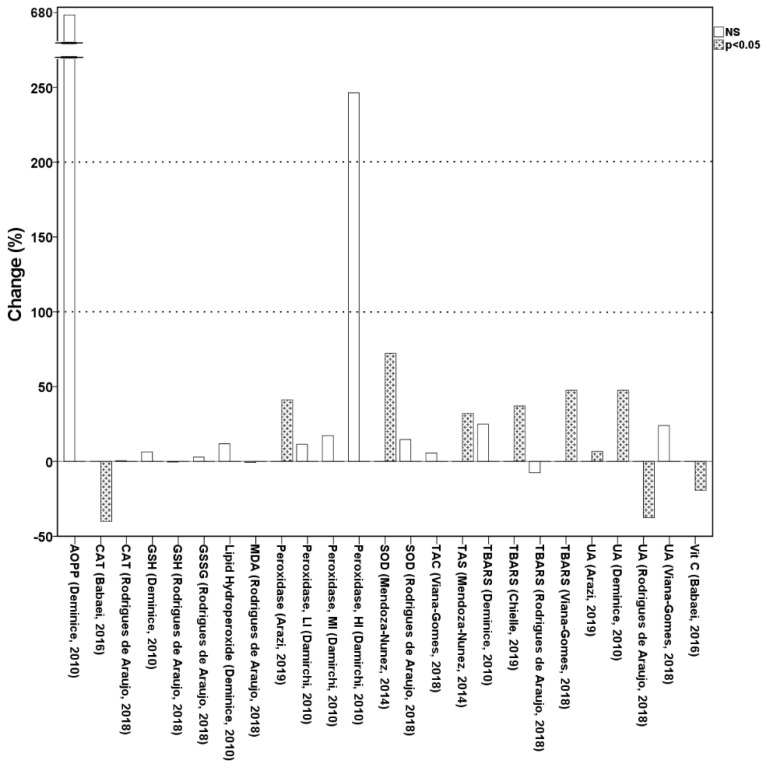
Salivary biomarkers of oxidative stress according to the studies included in the systematic review. Note: Data are reported as percentage changes after physical activity interventions and according to the significance level (i.e., NS = not significant). HI = high intensity of physical activity; MI = moderate intensity of physical activity; LI = low intensity of physical activity.

Salivary biomarkers followed very heterogeneous patterns, increasing or decreasing after physical activity (Figure 2). Refs. [29,30,31,34,45,58,68] reported significant changes due to physical activity. In [29], salivary peroxidase and UA were significantly increased in a group of non-smoker females (*n* = 12, age = 22.7 ± 2.9) who performed the Bruce protocol treadmill test until exhaustion. Participants were healthy and were abstaining from exercise for three months. Salivary SOD and TAS significantly increased after a six-month intervention of Thai Chi (5 days per week) involving 24 sedentary volunteers, aged between 60–74 years, who were diagnosed with periodontal disease [45]. [30] examined the effect of 1 h of exhaustive treadmill running on CAT and Vitamin C, finding a significant decrease in saliva shortly after exercise in 25 healthy sedentary males (21 ± 3 years). Salivary TBARS significantly increased in [31,68], both involving professional soccer players. [68] measured TBARS in salivary samples provided by eight athletes (males, 27.2 ± 5.5 years) after a 90 min soccer game. [31], instead, investigated TBARS changes in 27 males aged 22.5 ± 4.2, engaged in a supervised anaerobic training protocol. As previously observed for [29], UA has significantly increased also in 11 healthy and well-trained males aged 25.9 ± 2.8 years who completed an experimental resistance exercise protocol, after a rest period of 72 h [34]. In contrast, [58] reported that salivary UA decreased in 32 soccer players (21.2 ± 4.2 years) after the Bangsbo Sprint Test [70].

#### 3.1.4. The Effect of Physical Activity on Oxidative Stress Biomarkers in Urine

Refs. [27,28,32,35,36,39,41,42,43,44,46,47,48,49,51,53,54,55,57,59,60,61,63,66,67] reported significant oxidative stress changes due to physical activity. Oxidative stress biomarkers were generally increased after vigorous physical activity. Refs. [41,63] independently reported that urinary isoprostane levels diminished in sedentary subjects performing moderate-intensity aerobic exercise. Medina and colleagues examined the effect of 2-week aerobic training on 15 young triathletes from Spain, observing that urinary isoprostane decreased in males [44]. Low-intensity physical activity was associated with diminished isoprostane and 8-OH-dG levels in 24 sedentary subjects (20–50 years) [35]. Conversely, Samjoo and colleagues reported decreased isoprostane after 3 months of moderate-intensity training involving obese subjects, although no changes were observed in lean subjects [61]. Refs. [27,36,53,66] investigated urinary 8-OH-dG in subjects assigned to either moderate or high-intensity physical activity groups. Ref. [27] reported that only moderate exercise significantly decreased DNA oxidation products in 17 patients with colorectal cancer (58 ± 2 years). Ref. [66] observed similar results in 10 young healthy master runners, reporting lower levels of urinary 8-oxo-dG after moderate-intensity continuous exercise. In a randomised controlled trial [36] involving 70 healthy, Caucasian and untrained women (aged 60–75 years), 31 subjects assigned to the moderate-intensity exercise group showed a significant decrease in 8-OH-dG levels, while the high-intensity group showed the diametric opposite trend. [53] reported that urinary 8-OH-dG was slightly decreased in 28 elderlies following a 14-week whole-body resistance exercise-training program at moderate intensity. Accordingly, Nojima and colleagues reported that moderate-intensity exercise training reduces 8-OH-dG levels in 87 patients with type 2 diabetes mellitus over 12 months [51].

Refs. [32,39,42,43,46,47,48,49,54,55,57,59,60,67] reported increased oxidative stress biomarkers, including isoprostanes, DNA oxidation products, hydrogen peroxide, malondialdehyde and allantoin. [32] observed that urinary isoprostane increased after a 157.8-km-race in 8 professional-trained cyclists from Spain aged 25.7 ± 3.3 years. Accordingly, [59,67] reported that isoprostane levels rose after a long-distance race in 20 athletes, aged from 27 to 46 and in 24 runners (41.8 ± 6 years), respectively. Ref. [54] measured DNA oxidation products in 23 healthy males who underwent a 30-day vigorous exercise training consisting of 8–11 h of physical activity per day, six days per week. Refs. [55,60] independently observed that DNA oxidation products increased after vigorous multi-day races in young and healthy athletes. Ref. [42] found increased isoprostane in 98 sedentary young men (19–30 years old) engaged in acute isokinetic eccentric exercise. Ref. [43] investigated the responses of oxidative stress to a resistance training protocol involving twelve healthy males (21.3 ± 2.3 years) and observed that isoprostane was significantly higher after a 12-week resistance training. Ref. [49] found increased isoprostane in 20 healthy males (19–30 years) after the completion of a single running protocol. Similarly, in [57] a single resistance training session induced a 40% increase in urinary isoprostane in 8 healthy men (22.4 ± 2.0 years). Urinary MDA was significantly elevated after a submaximal exercise cycle ergometry test performed by 11 patients with COPD [46]. Similarly, [48] reported that urinary 8-OH-dG increased in 18 severe COPD patients (IV stage) who underwent pulmonary rehabilitation consisting of aerobic exercise training and education for 8 weeks. Only one study [39] among those reporting a significant increase of oxidative stress biomarkers, observed that urinary isoprostane and hydrogen peroxide were higher in 29 idiopathic pulmonary fibrosis patients after low-intensity bicycle challenges.

### 3.2. Quality of the Studies

Out of 43 studies, 25 (58%) were classified as “high quality”, comprising mainly before-after design (*n* = 16), controlled before-after (*n* = 5), RCTs (*n* =3) and randomised cross over (*n* = 1). On the contrary, 18 articles were identified as potentially affected by the risk of bias and accordingly classified as “medium quality”. They were based on a longitudinal design (*n* = 7), before-after or RCTs (*n* = 4, respectively), controlled before-after (*n* = 2) and case series self-controlled (*n* = 1). Specific quality domains were identified as potentially biased. In particular, in 90% of the studies with a before–after design, researchers assessing the outcomes were not blinded to the participant’s exposures or interventions. In 79% of the before-after studies, outcome measures of interest (i.e., oxidative stress biomarkers) were not taken multiple times before the intervention, whereas several studies measured oxidative stress biomarkers multiple times after the intervention. The majority of the observational studies (88%) did not provide any sample size justifications nor statistical power descriptions. None of the-observational studies examined physical activity at different levels (e.g., subgroups by intensity); however, it is worth mentioning that 63% of them analysed oxidative biomarker changes during competitive races. Subjects’ allocation was not concealed in the majority of the RCTs (88%) and 88% of the RCTs, there was no blinding of the subjects neither of the researchers who administered the training protocol.

### 3.3. Meta-Analyses of Exercise-Induced Oxidative Stress Biomarkers

**Figure 3:**
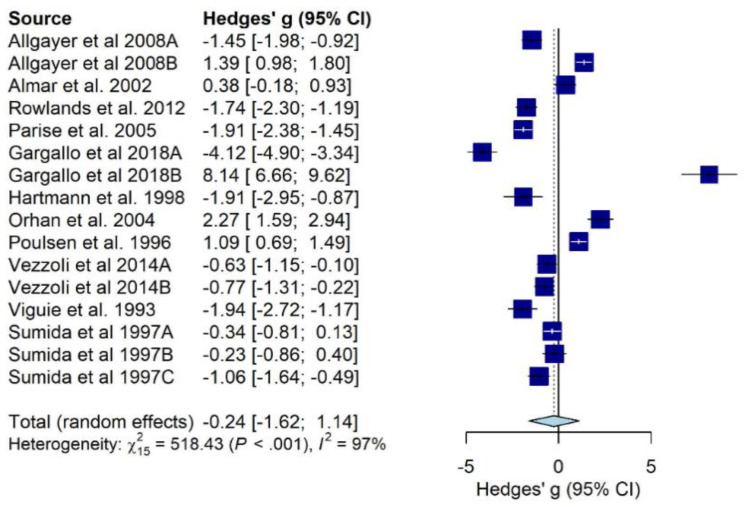
Pooled effect of physical activity interventions and oxidative stress measured by urinary 8-OH-dG or 8-oxo-dG. Note: Data presented as sub-groups “A” and “B” refer to moderate-intensity physical activity and high-intensity physical activity, respectively. Data presented as sub-groups “A”, “B” and “C” refer to running on a treadmill until exhaustion, cycling until exhaustion and running for 20 km, respectively.

**Figure 4:**
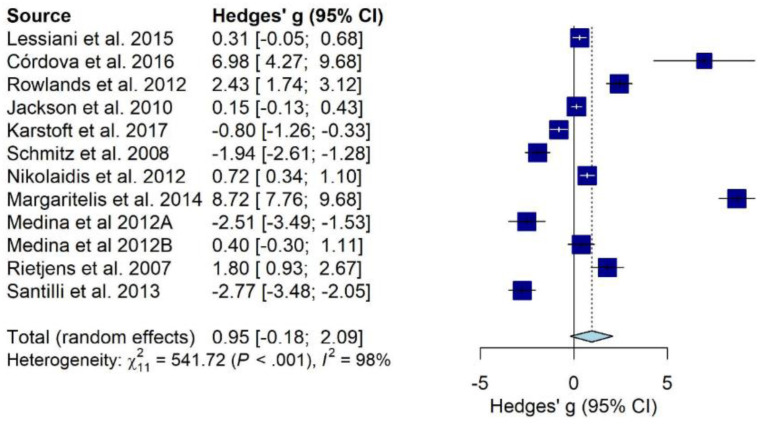
Pooled effect of physical activity interventions and oxidative stress measured by urinary isoprostanes. Note: Data presented as sub-groups “A” and “B” refer to males and females, respectively.

DNA oxidation products (Figure 3) were generally unchanged after physical activity (−0.24, 95% CI from −1.62 to 1.14), while isoprostanes (Figure 4) showed a trend of increase associated with physical activity (+0.95, 95% CI from −0.18 to 2.09).

Substantial heterogeneity was detected in both meta-analyses (I^2^ = 97%, *p* < 0.001; I^2^ = 98%, *p* < 0.001, respectively). Refs. [36,52] were identified as outliers and removed from DNA oxidation products meta-analysis and, although the heterogeneity remained very high (I^2^ = 95%, *p* < 0.001), physical activity was significantly associated with the decrease of 8-oxo-dG or 8-OH-dG (−0.68, 95% CI from −1.37 to 0.00). We explored the contribution of each study to the overall heterogeneity, detecting that [27,53,54] contributed the most in the meta-analysis on DNA oxidation products and [42] in the meta-analysis on isoprostanes. However, the subsequent leave-one-out analysis did not show any significant reductions in heterogeneity, even after removing those studies identified as greater contributors in terms of heterogeneity. No publication bias was found in any meta-analyses (*p* = 0.88 and *p* = 0.44, respectively) (Figures S1 and S2 in the supplementary material). Finally, several meta-regressions were carried out accounting for the potential effect of other predictors.

### 3.4. Meta-Regressions and Subgroup Analysis

#### 3.4.1. Meta-Regression Analyses on DNA Oxidation Biomarkers (8-oxo-dG or 8-OH-dG)

Study quality did not influence biomarkers changes (medium vs. high-quality β: +1.21, 95% CI from −1.61 to 4.04, *p* = 0.373) and physical activity types were not associated with any changes of DNA oxidation biomarkers (anaerobic versus aerobic (β: +0.80, 95% CI from −3.10 to 4.70, *p* = 0.666). No differences were detected in oxidative stress when comparing moderate and high-intensity physical activity (β: −1.45, 95% CI from −4.22 to 1.32, *p* = 0.280). We further inspected the subject’s training status influence on oxidative stress biomarker modulation, observing that nor being amateurishly trained (i.e., non-athlete), neither sedentary implied statistically significant differences in oxidative stress as compared to the athlete category (β: +0.28, 95% CI from −3.05 to 3.61, *p* = 0.857 and β: +1.60, 95% CI from −2.42 to 5.63, *p* = 0.404, respectively). Finally, the participants’ pathological condition did not influence exercise-induced DNA oxidation biomarkers, as no differences among healthy subjects compared to those reporting any pathologies, were found (β: −2.24, 95% CI from −4.56 to 4.07, *p* = 0.906).

#### 3.4.2. Meta-Regressions and Subgroup Analyses on Lipid Peroxidation Biomarkers (Isoprostanes)

Anaerobic physical activity determined a greater induction of urinary isoprostanes compared to aerobic exercise (β: +5.21, 95% CI from 2.76 to 7.66, *p* < 0.0001). Isoprostane was markedly reduced by both low and moderate intensities of physical activity compared to strenuous exercise levels (β: −5.73, 95% CI from −9.80 to −1.66, *p* = 0.0058 and β: −6.33, 95% CI from −8.92 to −3.73, *p* < 0.0001, respectively) and increased after vigorous physical activity (+6.01, 95% CI: 1.18; 10.84, *p* < 0.001). We observed that interventions protracted over time (e.g., weeks or months) were associated to a general decrease of urinary isoprostane (β: −1.19, 95% CI from −2.25 to −0.12, *p* < 0.001), while single exercise bouts or acute physical activity (e.g., hours or few days) determined the opposite (β: +3.29, 95% CI from 1.36 to 5.21, *p* < 0.001).

## 4. Discussion

Physical activity can prevent several non-communicable diseases [71] and contribute to ameliorating the quality of life [72]. These peculiar hallmarks strengthen the role of physical activity in public health and continue stimulating research efforts. Although modern redox biology has done great strides, the understanding of the modulation of oxidative stress by physical activity and exercise is still incomplete. Epidemiologic approaches and large-scale studies could support further research in this field; however, a general hint on one or more preferential biomarkers to quantify exercise-induced oxidative stress in non-invasive media is still lacking.

We observed that oxidation products and antioxidant species were the most frequently used physical activity-induced oxidative stress biomarkers in urine and saliva. The first group was preponderant in urine samples, covering a substantial percentage of the totality of the articles (more than 86%), while antioxidant species were predominantly quantified in saliva. Although saliva may represent an optimal non-invasive media, especially when dealing with a large population and/or uncooperative subjects, literature is scarce and no conclusive evidence can be drawn about salivary oxidative stress biomarkers quantification. Salivary biomarkers have shown an extremely heterogeneous pattern indicating augmentation, drop, or unchanged levels after physical activity even for the same biomarker (i.e., UA, TBARS) in different studies.

Conversely, there is plenty of literature on biomarkers quantified in urine. Urinary isoprostanes and DNA oxidation products were the two most frequently quantified biomarkers of oxidative stress. The association between DNA oxidation products and physical activity did not reach the significance level, discouraging any conclusive interpretations on 8-oxo-dG or 8-OH-dG modulation by exercise. Isoprostanes showed a general trend of the increase due to physical activity interventions, suggesting that changes in urinary isoprostanes might be successfully detected in urine after exercise; hence, isoprostanes might represent useful urinary biomarkers, although the weakness of the association and lack of homogeneity indicate that further research is needed.

Previous literature acknowledged physical activity as a potent inducer of lipid peroxidation, both in humans and in animals [73,74,75,76]. In a previous literature review [77], F2-IsoP, measured in plasma and skeletal muscles, increased after acute exercise, whereas urinary levels were generally increased but this trend required further confirmation. Similarly, [78] summarised the evidence from two studies reporting that isoprostane levels were increased after acute and intense exercise, either in skeletal muscles or in plasma, respectively. In another review [79], Sacheck and colleagues observed that plasma isoprostane levels were increased in horses after a treadmill test, were unaffected by 8 weeks of moderate/low exercise in subjects with type 2 diabetes, or even decreased in urine from trained rats after eccentric muscle exercise. On the contrary, a recent systematic review [71] stated that plasmatic isoprostane is generally reduced after an exercise-training period in both elderly and young subjects and the same result was observed for urinary isoprostane, but only when accompanied by relatively marked gains in aerobic fitness.

Our findings highlighted that anaerobic physical activity induced a greater increase of urinary isoprostanes than aerobic exercise. Although, out of 11 studies, only [57] and [42] applied anaerobic interventions with medium and high intensity, respectively. Noticeably, also aerobic interventions determined an oxidative stress augmentation, which was smaller than that observed after anaerobic exercise and exclusively related to high-intensity aerobic exercise protocols.

In terms of physical activity intensity, our findings suggest that urinary isoprostanes were markedly reduced after both low and moderate intensity of exercise compared to strenuous physical activity. Previous literature reviews suggest similar results: high intense and prolonged aerobic exercise [80] and even anaerobic exercise [81,82,83], have been associated with greater ROS production, thus, oxidative stress [84].

Urinary isoprostanes were generally reduced after physical activity interventions protracted over time, suggesting that regular exercise training could act as an antioxidant at least against isoprostane formation in vivo. On the contrary, we observed that isoprostanes augmented after an acute bout of physical activity, supporting the hypothesis that physical activity could act also as a stressor. These findings are in line with the review published by Nikolaidis in 2011, who reported for the first the comparison between acute and chronic exercise on isoprostanes measured in three different specimens: plasma, urine and skeletal muscle [77]. The underlying mechanism invokes the in vivo upregulation of antioxidant levels, which can be enhanced by regular exercise training that acts as an adaptive *stimulus* [75,85].

The physiological mechanisms supporting the general finding that oxidative stress can increase after physical activity are fairly well-accepted and understood. During physical activity, several pathways are involved in ROS overproduction: (1) aerobic metabolism and electron chain, which increases the leakage of superoxide radicals due to the increased oxygen consumption while exercising; (2) anaerobic exercise, in turn, can activate different pathways including NADPH oxidase, xanthine oxidase, ischemia-reperfusion, purine oxidation and catecholamine auto-oxidation [81,86].

A general remark should be outlined as each oxidative stress biomarker presents advantages and disadvantages [75] and can be affected by a multitude of other factors, including physical activity type and duration [75] as well as the timing of sampling [87] and inter-individual variability [88]. All these aspects have been conducted to a general and well-supported indication mainly referring to the assessment of a set of biomarkers instead of a preferential one. Therefore, although our results slightly support the quantification of isoprostanes in urine when dealing with exercise-induced oxidative stress investigations, we recommend the most integrative approach of multi-marker panels. Urine remains a suitable non-invasive medium with low metal and organic content and whose collection is easy and cost-effective [18,19,89]; however, biomarkers quantification deserves further harmonisation in terms of sampling timing, type of specimen (spot, 12 h and 24 h urine), normalisation to creatinine, analytical method and statistical reporting of results.

To reduce possible drawbacks during the sampling and handling of urinary samples we suggest the following recommendations. First, each subject’s clinical status must be investigated, since renal and/or bladder impairments together with other pathologies able to indirectly affect renal/bladder functionality (e.g., diabetes), can contribute to local biomarker formation, altering the total amount attributable to other sites. Second, creatinine needs to be quantified to normalise spot urines, to evaluate kidney efficiency and to reduce intra and inter variability among subjects. A third point should be extended to all biological samples, which all requires a careful control of the temperature during sampling, handling and long-term storage to avoid auto-oxidation. In addition, comparative studies, focusing on different analytical methods, could provide specific corrective factors to support the harmonisation between different analytical techniques. Since, beyond the specimen characteristics, the paramount diversity in analytical techniques and nomenclature systems adopted by different laboratories hamper comparisons among studies. Although chemical techniques are considered superior to immunological techniques based on their higher sensitivity and specificity [90], they are time-consuming and expensive. Therefore, ELISA are becoming more popular, as confirmed from this systematic review. Irrespective from ELISA’s poor correlation with chemical assays and their lower specificity, they are cost-effective and may support epidemiological and large-scale studies.

Future development in exercise-induced oxidative stress evaluation in non-invasive media should take into account: (1) emerging media such as saliva, which is a promising non-invasive medium that still requires protocols standardisation [91] in terms of sampling timing and analysis protocols and techniques; (2) physical activity duration, intensity and type as well as sample collection timing, handling and storage can considerably affect as ROS induction as oxidative stress quantification [19]; (3) the intra-individual variability imposes multiple time points assessment of oxidative stress biomarkers, especially at baseline (i.e., before physical activity intervention); (4) low sample sizes, non-blinded researchers and non-concealed allocation of the participants enrolled in randomised trials should be avoided and (5) the quantification of exercise-induced oxidative stress biomarkers should be always complemented with the anti-oxidant counterparts, which is intimately linked to oxidative status and strongly affected by physical activity itself.

As a main strength, we acknowledge that the present study systematically reviews and meta-analyses, for the first time, exercise-induced oxidative stress biomarkers in non-invasive media. The included studies were generally scored highly in terms of quality; thus, the overall risk of bias was low. One of the main limitations is the substantial heterogeneity that has been observed in the meta-analyses. Although we performed a set of sensitivity analyses, none of them were able to elucidate such a heterogeneity; thus, meta-analysis results should be taken with cautiousness. Therefore, we recommend an integrative approach that involves multi-marker panels of oxidative stress to accurately assess the effect of physical activity on oxidative stress levels.

## 5. Conclusions

Despite the wide heterogeneity among a large set of oxidative stress biomarkers quantified in both urine and saliva, the present meta-analysis concluded that urinary isoprostanes seem more prone to physical activity modulation. Indeed, we observed that physical activity could elicit an increase of urinary isoprostanes that is greater after anaerobic exercise compared to aerobic one. In addition, low to moderate physical activity seems to evoke a reduction of urinary isoprostanes compared to strenuous exercise as observed for long-lasting training versus single acute bouts. Conversely, no conclusive results have been observed nor for DNA oxidation products quantified in urine after physical activity, for other oxidative stress biomarkers quantified both in urine and in saliva.

## Figures and Tables

**Table 1:** Study and participants characteristics as originally reported by the studies included in the systematic review. Quality assessment is study-design based.

Study	Country	Design	Sample Population (*n*; Sex; Age; Training; Health)	Physical Activity (Type; Duration; Intensity)	Main Findings	Quality Assessment	Ref.
Allgayer 2008	Germany	RCT	♂ 17 moderately active, age (SD) = 58 (2) ♀ 27 active, age (SD) = 59 (1) non-athlete, cancer patients	aerobic 35 min duration 30–60% Vo2max	↑ Urinary 8-OH-dG. After 2 weeks of individualised aerobic exercise (30–40 min/Day)	High	[27]
Almar 2002	Spain	Longitudinal	♂ 8 Age (SD) = 25.5 (1.7) athlete, healthy	mixed NA duration 75% Vo2max	N.S. changes in urinary 8-OH-dG normalised to creatinine. ↑ not normalised 8-OH-dG. After a 3-day cycling race	Medium	[28]
Arazi 2019	Iran Japan	Controlled before-after	♀ 23 11 smokers, age (SD) =23.6 (2.9) 12 non-smokers, age (SD) = 22.7 (2.9) sedentary, healthy	aerobic NA duration Until exhaustion	↑ Salivary peroxidase ↑ Salivary UA (non-smokers). After a treadmill bout until exhaustion	High	[29]
Babaei 2016	Iran	RCT	♂ 25 Age (SD) = 21 (3) sedentary, healthy	aerobic NA duration Until exhaustion	↓ Salivary CAT, Vitamin C. After treadmill run	Medium	[30]
Chielle 2019	Brazil	Before after	♂ 27 Age (SD) = 22.5 (4.2) athlete, healthy	mixed 60 min duration NA intensity	↑ Salivary TBARS ↑ TBARS After supervised training	High	[31]
Córdova 2015	Spain	Longitudinal	♂ 8 Age (SD) = 25.7 (3.3) athlete, healthy	aerobic 230 min duration 85% Vo2max	↑ Urinary isoprostane. After a cycling race	Medium	[32]
Damirchi 2010	Iran	Controlled before-after	♂ 10 Age (SD) = 23.2 (2.3) non-athlete, healthy	aerobic 18 min duration 50–75% VO2max	↑ Salivary peroxidase. After a treadmill run until exhaustion	Medium	[33]
Deminice 2010	Brazil	Before after	♂ 11 Age (SD) = 25.9 (2.8) non-athlete, healthy	anaerobic NA duration 75% 1RM	↑ Salivary UA. N.S. changes in TBARS, AOPP, GSH and lipid hydroperoxides After resistance exercise	High	[34]
Devries 2008	Canada	Controlled before-after	♀ 24 12 lean, age (SD) = 41 (2) 12 obese, age (SD) = 40 (3) sedentary, healthy and obese	aerobic 38 min duration 50–65% Vo2max	↓ Urinary 8-OH-dG ↓ Isoprostane After a 12-week program of endurance training	High	[35]
Gargallo 2018	Spain	RCT	♀ 36 High intensity, age (SD) = 71.1 (5.3) Moderate intensity, age (SD) = 68.7 (6.1) sedentary, healthy	anaerobic 58 min duration 70–85% Vo2max	↑ Urinary 8-OH-dG and ↓ Urinary 8-OH-dG, after high and moderate exercise intensity, respectively	Medium	[36]
Hartmann 1998	Austria Germany	Before after	♂ 3 ♀ 3 Age (SD) = 27 (6) athlete, healthy	aerobic 150 min duration High intensity (Triathlon)	↑ Urinary 8-OH-dG. After a 24 h short-distance triathlon	High	[37]
Hofer 2008	Italy USA	RCT	♂ 5 ♀12 Age (SD) = 58.6 (2.7) sedentary, healthy	aerobic NA duration NA intensity	Urinary nucleic acid oxidation products were not significantly different from baseline following 12-month intervention program	Medium	[38]
Jackson 2010	USA	RCT	♂ 23 ♀ 6 Age (SD) = 70 (9) sedentary, idiopathic pulmonary fibrosis	aerobic 4 min duration low intensity (50-W bicycle)	↑ Urinary isoprostane, N.S. changes in urinary H_2_O_2_ found after 50-W bicycle	High	[39]
Karstoft 2017	Denmark UK	Randomised crossover trial	♂ 11 ♀ 3 Age (SD) = 65 (2) non-athlete, diabetic	aerobic 60 min duration 75% Vo2max	N.S. changes in urinary isoprostane after 60 min of supervised waling on a treadmill	High	[40]
Lessiani 2016	Italy	Before after	♂ 12 ♀ 6 Age (IQR) = 54 (48–66) sedentary, healthy	aerobic 55 min duration 75% Vo2max	↓ Urinary isoprostane. After an eight-week aerobic training program	High	[41]
Margaritelis 2014	Cyprus Greece	Before after	♂ 98 age (SD) = 23.5 (4) non-athlete	anaerobic NA duration high intensity (maximal voluntary contractions)	↑ Urinary isoprostane 48 h after an acute isokinetic eccentric exercise bout	High	[42]
Margonis 2007	Greece	Before after	♂ 12 age (SD) = 22.4 (2.1) non-athlete, healthy	anaerobic NA duration 85% Vo2max	↑ Urinary isoprostane 96 h after a 12-week resistance training protocol	Medium	[43]
Medina 2012	Spain	Longitudinal	♂ 10 Age (SD) = 19.0 (1.7) ♀ 5 Age (SD) = 21.8 (3.0) athlete, healthy	aerobic NA duration NA intensity	↓ Urinary total isoprostanes and 8-iso-15-keto PGF2α in males after 3 weeks of triathlon training. N.S. changes in isoprostane, 8-iso-15(R) PGF2α, 2,3-dinor-8-iso PGF2α, 2,3-dinor-11β PGF2α	Medium	[44]
Mendoza-Núñez 2014	Mexico	Before after	♀ 24 Age (SD) = 67 (7) sedentary, periodontal disease	aerobic 60 min duration 55% Vo2max	↑ Salivary SOD and TAS ↓ IL-1β. After 6 months of Tai Chi 5 days/week	High	[45]
Mercken 2005	Netherlands	Controlled before-after	♂ 11 ♀ 11 11 healthy, age (SD) = 59.7 (1.5) 11 COPD patients, age (SD) = 56.7 (2.0) sedentary, non-athlete, healthy and COPD	aerobic 23 min duration 60% Vo2max	↑ Urinary MDA in COPD patients shortly after submaximal exercise and maximal exercise before pulmonary rehabilitation	High	[46]
Mikami 2000	Japan	Controlled before-after	♂ 7 Age range = 20–30 non-athlete, healthy	aerobic 40 min duration 40–100% VO2max	↑ Urinary allantoin shortly after moderate intensity cycling exercise. N.S. changes of urate nor TBARS	High	[47]
Nemoto 2014	Japan	Before after	♂ 18 12 COPD II and III stage, age (SD) = 71, (1.3) 6 COPD IV stage, age (SD) = 65.7(1.52) COPD	aerobic 20 min duration 70% Vo2max	↑ Urinary 8-OH-dG in COPD (Stage IV) following 8-week pulmonary rehabilitation and aerobic training	High	[48]
Nikolaidis 2012	Cyprus Greece	Before after	♂ 20 10 muscle damaging, age (SD) = 27 (6) 10 no muscle damaging, age (SD) = 25 (5) non-athlete, healthy	aerobic 45 min duration 70–75% Vo2max	↑ Urinary isoprostane shortly after a running protocol on a treadmill	High	[49]
Nikolaidis 2013	Cyprus Greece	Before after	♂ 20 10 young, age(SD) = 20.6 (0.5) 10 elderly, age (SD) = 64.6 (1.1) non-athlete, healthy	anaerobic NA duration 30–50% Vo2max	↑ Urinary isoprostane in elderly shortly after a single bout of isokinetic eccentric exercise	High	[50]
Nojima 2008	Japan	RCT	♂ 59 ♀ 28 Exercise in a fitness centre, age (SD) = 55.4 (1.1) Self-paced exercise, age (SD) = 55.9 (1.1) non-athlete, diabetic	aerobic 30 min duration 50% Vo2max	↓ Urinary 8-OH-dG after 12-month program of aerobic exercise either self-paced either in a fitness centre	High	[51]
Orhan 2004	Netherlands	Case series self-controlled	♂ 18 Age (SD) = 24.6 (0.7) non-athlete, healthy	aerobic 60 min duration 70% Vo2max	↑ Urinary 8-OH-dG 1 day after 1 h cycling bout (*p* = 0.07) N.S. changes of urinary MDA	Medium	[52]
Parise 2005	Canada	Before after	♂ 15 ♀ 15 Age (SD) = 68.5 (5.1) non-athlete, healthy	anaerobic NA duration 65% 1RM	↓ Urinary 8-OH-dG. After a 14-week program of resistance training	High	[53]
Poulsen 1996	Denmark	Before after	♂ 23 Age (SD) = 22 (2) athlete, healthy	aerobic 570 min duration high intensity (30-day program of vigorous exercise)	↑ Urinary 8-OH-dG after a 30-day program of vigorous exercise (6 days per week, 8–11 h per day)	Medium	[54]
Radák 2000	Canada Hungary	Longitudinal	♂ 5 Age (SD) = 35.5 (9.5) athlete, healthy	aerobic NA duration high intensity (marathon)	↑ Urinary 8-OH-dG after the first day (120 km) of a 4-day race (marathon)	Medium	[55]
Rall 2000	USA	Controlled before-after	♂ 6 ♀ 10 8 healthy, age (SD) = 70.3 (5.0) 8 arthritis rheumatoid, age (SD) = 41.8 (12.6) sedentary, healthy and rheumatoid arthritis	anaerobic NA duration 80% 1 RM	N.S. changes in urinary 8-OH-dG following a 12-week progressive resistance training	Medium	[56]
Rietjens 2007	Netherlands	Before after	♂ 8 Age (SD) = 22.4 (2) athlete, healthy	anaerobic 41 min duration 75% Vo2max	↑ Urinary isoprostane following a single session of resistance exercise	Medium	[57]
Rodrigues de Araujo 2018	Brazil	Before after	♂ 32 Age (SD) = 21.2 (4.2) healthy	anaerobic 3 min duration high intensity	↓ Salivary UA and N.S. changes in salivary TBARS, MDA, GSH, GSSG, SOD and CAT after acute high intensity exercise	High	[58]
Rowlands 2012	Canada	Longitudinal	♂ 16 ♀ 3 Age (SD) = 37.0 (6.7) athlete, healthy	aerobic 5700 min duration high intensity (894-Km run)	↑ Urinary isoprostane N.S. changes in 8-0H-dG. After an 894-km run	Medium	[59]
Samia 2014	Egypt	Longitudinal	♀ 8 Age (SD) = 22.9 (4.2) athlete	mixed 150 min duration high intensity (National 1st Class Republic Competition)	↑ Urinary 8-OH-dG after the first day of the National First, Class Republic Competition consisting of 100 m run, high jump, shot put and 200 m run.	Medium	[60]
Samjoo 2013	Canada USA	Before after	♂ 18 9 lean, age (SD) = 38 (3) 9 obese, age (SD) = 39 (3) sedentary, healthy and obese	aerobic 45 min duration 50–70% Vo2max	↓ Urinary isoprostane and ↑ Urinary 8-OH-dG (*p* = 0.07) in obese after 3-month endurance cycling training	High	[61]
Santilli 2013	Italy	Before after	♂ 15 ♀ 7 Age (SD) = 57 (9) sedentary, healthy	aerobic 50 min duration 65% Vo2max	↓ Urinary isoprostane after 8-week aerobic training program	High	[62]
Schmitz 2008	USA	Before after	♀ 15 Age (SD) = 20.9 (2.4) sedentary, healthy	aerobic 30 min duration 70–85% Vo2max	↓ Urinary isoprostane after 15 weeks of aerobic exercise	High	[63]
Sumida 1997	Japan	Controlled before-after	♂ 28 11 runners’ group I, age (SD) = 20.7 (0.5) 6 untrained, age (SD) = 9.8 (0.3) 11 runners’ group II, age (SD) = 19.1 (0.2) athletes, sedentary, healthy	aerobic NA duration high intensity	N.S. changes in urinary 8-OH-dG after a single bout of intensive exercise	High	[64]
Vempati 2009	India	RCT	♂ 13 ♀ 16 Age (SD) = 33.5 (11.4) sedentary, asthmatic patients	aerobic 240 min duration low intensity (yoga)	N.S. changes in urinary 11β-PGF2α after 8-week yoga intervention	Medium	[65]
Vezzoli 2014	Italy	Before after	♂ 20 moderate-intensity training, age (SD) = 50.6 (6.3) high-intensity training, age (SD) = 45.1 (8.5) healthy	aerobic NA duration 80–140% VO2peak	↑ Urinary 8-OH-dG after either high-intensity discontinuous training and moderate-intensity continuous training	High	[66]
Vezzoli 2016	Italy	Longitudinal	♂ 10 ♀ 14 50 km race group, age (SD) = 41.8 (5.9) 100 km race group, age (SD) = 41.4 (3.6) athlete, healthy	aerobic 426 min duration high intensity (ultra-endurance exercise)	↑ Urinary isoprostane and 8-OH-dG after ultra-endurance exercise	Medium	[67]
Viana-Gomes 2018	Brazil	Before after	♂ 8 Age (SD) = 27.2 (5.5) athlete, healthy	mixed 64 min duration high intensity (Football game)	N.S. changes in salivary TAC, UA. ↑ Salivary TBARS. After one football game	High	[68]
Viguie 1993	USA	Before after	♂ 11 Age (SD) = 24.3 (1.1) healthy	aerobic 90 min duration 65% Vo2max	N.S. changes of urinary 8-OH-dG following single exercise bout on a cycle ergometer	Medium	[69]

♂: males; ♀: females; ↑: increase; ↓; decrease; NA: Not Available; RCT: Randomised Controlled Trial; VO2max: maximal oxygen consumption; Vo2peak: peak oxygen uptake; RM: Repetition Maximum; W: Watts, cycling power.

**Table 2:** Biomarkers of oxidative stress measured in urine and/or saliva at baseline and after physical activity intervention. Data are presented as originally reported by the studies as follows: * mean (SEM), ^†^ median (IQR), ^§^ mean (SD).

Study	Sample	Urinary Biomarker	Analytical Method	Urinary Baseline Measure	After Physical Activity	Salivary Biomarker	Analytical Method	Salivary Baseline Measure	After Physical Activity	Ref.
Allgayer 2008	12 h	8-oxo-dG [ng/mg crea]	HPLC	moderate intensity 8.5 (2.0) ^§^ high intensity 5.0 (1.3) ^§^	5.8 (1.5) ^§^ 7.1 (1.6) ^§^	--	--	--	--	[27]
Almar 2002	12 h	8-OH-dG [nmol/mmol crea]	HPLC	117.4 (38.1) *	136.5 (54.8) *	--	--	--	--	[28]
Arazi 2019	spot	--	--	--	--	UA [mg/100 mL]	Spectrophotometric (enzymatic reaction)	non-smokers 5.9 (0.8) * smokers 5.8 (0.6) *	6.3 (0.8) * 5.9 (0.7) *	[29]
Arazi 2019	spot	--	--	--	--	Peroxidase [mm/u]	Colorimetric	non-smokers 0.17 (0.07) * smokers 0.13 (0.08) *	0.24 (0.08) * 0.19 (0.08) *	
Babaei 2016	spot	--	--	--	--	CAT [u/mL]	Spectrophotometric	0.005 (0.001) ^§^	0.003 (0.001) ^§^	[30]
Babaei 2016	spot	--	--	--	--	Vitamin C [mg%]	Colorimetric	0.274 (0.29) ^§^	0.221 (0.45) ^§^	
Chielle 2019	spot	TBARS [mmol/L]	ELISA	13.6 (7.3) ^§^	80.1 (14.3) ^§^	TBARS [mmol/L]	ELISA	19.4 (11.7) ^§^	26.6 (18) ^§^	[31]
Córdova 2015	spot	isoprostane [pg/mg crea]	ELISA	359 (71) ^§^	686 (139) ^§^	--	--	--	--	[32]
Damirchi 2010	spot	--	--	--	--	Peroxidase [U/L]	Spectrophotometric	50% VO2max 3.22 (0.24) ^§^ 75% VO2max 3.47 (0.37) ^§^ Exhaustion 3.17 (0.40) ^§^	3.59 (0.25) ^§^ 4.07 (0.38) ^§^ 10.98 (0.27) ^§^	[33]
Deminice 2010	spot	--	--	--	--	TBARS [umol/L]	Colorimetric (Ellman’s reaction)	2.0 (1.2) ^§^	2.5 (1.2) ^§^	[34]
Deminice 2010	spot	--	--	--	--	Lipid hydroperoxide [umol H_2_O_2_ equivalents/L]	Colorimetric	10.2 (2.6) ^§^	11.4 (4.5)v	
Deminice 2010	spot	--	--	--	--	AOPP [umol chloromina T equivalents/L]	Spectrophotometric	30.8 (14.8) ^§^	37.4 (17.7) ^§^	
Deminice 2010	spot	--	--	--	--	UA [mg/dL]	Enzymatic	2.1 (1.1) ^§^	3.1 (1.1) ^§^	
Deminice 2010	spot	--	--	--	--	GSH [umol/L]	Colorimetric (Ellman’s reaction)	0.16 (0.08) ^§^	0.17 (0.08) ^§^	
Devries 2008	24 h	isoprostane [pg/mL]	ELISA	lean 54.41 (17.12) * obese 72.20 (17.97) *	37.97 (8.3) * 53.22 (12.03) *	--	--	--	--	[35]
Devries 2008		8-OH-2-dG [ng/mL]	ELISA	lean 5.71 (1.31) * obese 15.02 (3.73) *	3.15 (0.76) * 4.69 (1.54) *	--	--	--	--	
Gargallo 2018	spot	8-OH-dG [nmol/mmol crea]	HPLC	high intensity 2.12 (1.34) ^§^ moderate intensity 3.91 (1.40) ^§^	3.64 (1.37) ^§^ 2.90 (1.54) ^§^	--	--	--	--	[36]
Gargallo 2018		GSH [nmol/mg protein]	Colorimetric (enzymatic recycling)	high intensity 22.71 (3.83) ^§^ moderate intensity 20.84 (3.15) ^§^	20.23 (3.35) ^§^ 20.69 (4.22) ^§^	--	--	--	--	
Gargallo 2018		GSSG [nmol/mg protein]	Colorimetric (enzymatic recycling)	high intensity 0.23 (0.08) ^§^ moderate intensity 0.25 (0.07) ^§^	0.25 (0.13) ^§^ 0.23 (0.09) ^§^	--	--	--	--	
Gargallo 2018		GSSG/GSH [%]	Colorimetric (enzymatic recycling)	high intensity 1.05 (0.48) ^§^ moderate intensity 1.25 (0.40) ^§^	1.29 (0.79) ^§^ 1.19 (0.57) ^§^	--	--	--	--	
Hartmann 1998	24 h	8-OH-dG [umol/mol crea]	HPLC	2.42 (1.26) ^§^	1.30 (0.23) ^§^	--	--	--	--	[37]
Hofer 2008	12 h	FapyGua [nmol/mmol crea]	MS-MS	4.50 (2.4) *	3.25 (1.0) *	--	--	--	--	[38]
Hofer 2008		8-oxoGua [nmol/mmol crea]	MS-MS	127 (28) *	144 (44) *	--	--	--	--	
Hofer 2008		8-oxoGuo [nmol/mmol crea]	MS-MS	6.28 (2.1) *	5.34 (1.5) *	--	--	--	--	
Hofer 2008		8-oxodGuo [nmol/mmol crea]	MS-MS	2.30 (0.74) *	2.78 (0.82) *	--	--	--	--	
Jackson 2010	spot	isoprostane [pg/g crea]	HPLC-MSMS	275 (184) ^†^	335 (295) ^†^	--	--	--	--	[39]
Jackson 2010		H_2_O_2_ [umol/mg crea]	Colorimetric	30.8 (15.4) ^†^	38.5 (53.8) ^†^	--	--	--	--	
Karstoft 2017	24 h	isoprostane [pg/mg crea]	ELISA	1148 (127) *	1051 (114) *	--	--	--	--	[40]
Lessiani 2016	12 h	isoprostane [pg/mg crea]	RIA	320 (287–435) ^†^	209 (154–258) ^†^	--	--	--	--	[41]
Margaritelis 2014	spot	isoprostane [pg/ng crea]	ELISA	690 (220) ^§^	950 (320) ^§^	--	--	--	--	[42]
Margonis 2007	spot	isoprostane [ng/mL]	ELISA	1.65 (1.43;1.89) *	3.90 (3.60;4.35) *	--	--	--	--	[43]
Medina 2012	24 h	Total isoprostanes [ng/24 h]	UPLC	males 12,920 (4790) ^§^ females 7700 (2900) ^§^	9380 (2910) ^§^ 8230 (1070) ^§^	--	--	--	--	[44]
Medina 2012		isoprostane [ng/24 h]	UPLC	males 1714.3 (723.7) ^§^ females 1476.2 (951.8) ^§^	1009.5 (485.5) ^§^ 809.5 (389.5) ^§^	--	--	--	--	
Medina 2012		8-iso-15(R)-PGF2α [ng/24 h]	UPLC	males 634.2 (451.2) ^§^ females 1341.5 (134.1) ^§^	1939 (841) ^§^ 902.4 (341.5) ^§^	--	--	--	--	
Medina 2012		2,3-dinor-8-iso PGF2α [ng/24 h]	UPLC	males 4000 (875) ^§^ females 2087 (725) ^§^	2887 (587.4) ^§^ 2824 (738) ^§^	--	--	--	--	
Medina 2012		2,3-dinor-11β-PGF2α [ng/24 h]	UPLC	males 3124.9 (1205) ^§^ females 2375 (772) ^§^	2295 (682) ^§^ 3454 (432) ^§^	--	--	--	--	
Mendoza-Núñez 2014	spot	--	--	--	--	SOD [UI/L]	Colorimetric	1.62 (0.83) *	2.79 (1.6) *	[45]
Mendoza-Núñez 2014	spot	--	--	--	--	TAS [mmol/L]	Colorimetric	0.53 (0.33) *	0.70 (0.35) *	
Mendoza-Núñez 2014	spot	--	--	--	--	TNF-alpha [pg/mL]	Flow cytometry	0.5119 (0.009) *	4.2410 (0.435) *	
Mendoza-Núñez 2014	spot	--	--	--	--	IL-1 beta [pg/mL]	Flow cytometry	783.62 (174.9) *	624.97 (196.7) *	
Mendoza-Núñez 2014	spot	--	--	--	--	IL-6 [pg/mL]	Flow cytometry	18.66 (7.25) *	4.76 (1.93) *	
Mendoza-Núñez 2014	spot	--	--	--	--	IL-8 [pg/mL]	Flow cytometry	4971.2 (835.0) *	2242.4 (330.0) *	
Mendoza-Núñez 2014	spot	--	--	--	--	IL-10 [pg/mL]	Flow cytometry	0.21 (2.5) *	2.9 (1.5) *	
Mendoza-Núñez 2014	spot	--	--	--	--	Lipid hydroperoxidase [umol/L]		0.11 (0.07) ^§^	0.14 (0.09) ^§^	
Mercken 2005	spot	MDA [μmol/mmol crea]	HPLC	healthy subjects 0.28 (0.04) * COPD patients 0.38 (0.02) *	0.29 (0.03) * 0.52 (0.07) *	--	--	--	--	[46]
Mikami 2000	spot	Allantoin [μmol/mg crea]	HPLC	40% VOmax 0.08 (0.002) * 100% VO2max 0.08 (0.001) *	0.09 (0.001) * 0.08 (0.001) *	--	--	--	--	[47]
Mikami 2000		TBARS [μmol/mg crea]	HPLC	40% VO2max 2.80 (0.8) * 100% VO2max 2.90 (0.4) *	2.76 (0.6) * 2.57 (0.4) *	--	--	--	--	
Mikami 2000		Urate [μmol/mg crea]	UA B-test Wako	40% VO2max 0.40 (0.06) * 100% VO2max 0.42 (0.06) *	0.40 (0.06) * 0.37 (0.07) *	--	--	--	--	
Nemoto 2014	spot	8-OH-dG [ng/mg crea]	ELISA	II–III COPD severity 16.6 (2.2) * IV COPD severity 14.6 (1.8) *	17.8 (2.3) * 24.3 (2.6) *	--	--	--	--	[48]
Nikolaidis 2012	spot	isoprostane [pg/ng crea]	ELISA	muscle damaging 588.8 (315.4) * non-muscle damaging 352.8 (196.3) *	1126.17 (324.78) * 967.29 (233.64) *	--	--	--	--	[49]
Nikolaidis 2013	spot	isoprostane [pg/mg crea]	ELISA	young 430.4 (30.4) * elederly 560.9 (39) *	434.8 (39) * 587 (47) *	--	--	--	--	[50]
Nojima 2008	spot	8-OH-dG [ng/mg crea]	ELISA	Exercise in a fitness centre 10.3 (1.1) * Self-paced exercise 11.3 (1.4) *	9.3 (1.0) * 8.1 (0.8) *	--	--	--	--	[51]
Orhan 2004	24 h	8-OH-dG [nmol/12 h]	ELISA	12.14 (5) *	47.4 (15) *	--	--	--	--	[52]
Orhan 2004		MDA [nmol/12 h]	HPLC	1.45 (0.33) *	1.74 (0.35) *					
Parise 2005	spot	8-OH-dG [ng/g crea]	ELISA	10783 (5856) ^§^	8897 (4030) ^§^	--	--	--	--	[53]
Poulsen 1996	spot	8-OH-dG [nmol/mmol crea]	HPLC	1.03 (0.59) ^§^	1.25 (0.59) ^§^	--	--	--	--	[54]
Radák 2000	spot	8-OH-dG [ng/mL]	ELISA	14.74 (2.50) ^§^	19.15 (2.50) ^§^	--	--	--	--	[55]
Rall 2000	24 h	8-OH-dG [nmol/day]	ELISA	healty elderly 24.82 (16.35) ^§^ rheumatoid arthritis 45.43 (16.67) ^§^	15.50 (10.74) ^§^ 30.11 (31.17) ^§^	--	--	--	--	[56]
Rietjens 2007	spot	isoprostane [nmol/mmol crea]	ELISA	0.117 (0.021) *	0.164 (0.030) *	--	--	--	--	[57]
Rodrigues de Araujo 2018	spot	--	--	--	--	TBARS [nmol/mL]	Colorimetric	9.20 (3.13) ^§^	8.50 (2.43) ^§^	[58]
Rodrigues de Araujo 2018	spot	--	--	--	--	MDA [uM]	Colorimetric	5.40 (2.15) ^§^	5.37 (1.52) ^§^	
Rodrigues de Araujo 2018	spot	--	--	--	--	GSH [uM]	Colorimetric	54.78 (3.57) ^§^	54.55 (9.57) ^§^	
Rodrigues de Araujo 2018	spot	--	--	--	--	GSSG [uM]	Colorimetric	2.04 (1.18) ^§^	2.10 (1.13) ^§^	
Rodrigues de Araujo 2018	spot	--	--	--	--	UA [ug/dL]	Colorimetric	2.66 (1.33) ^§^	1.66 (0.92) ^§^	
Rodrigues de Araujo 2018	spot	--	--	--	--	SOD [U/g dL^−1^]	Spectrophotometric	32.6 (43.9) ^§^	37.4 (42.1) ^§^	
Rodrigues de Araujo 2018	spot	--	--	--	--	CAT [U/g dL^−1^]	Colorimetric	1.65 (1.53) ^§^	1.66 (2.90) ^§^	
Rowlands 2012	spot	isoprostane [pg/umol crea]	ELISA	84.9 (28.6) ^§^	112.6 (52.7) ^§^	--	--	--	--	[59]
Rowlands 2012		8-OH-dG [pg/umol crea]	ELISA	11166 (5613) ^§^	9045 (4813) ^§^	--	--	--	--	
Samia 2014	spot	8-OH-dG [nmol/L]	ELISA	23.78 (1.95) ^§^	25.96 (1.33) ^§^	--	--	--	--	[60]
Samjoo 2013	24 h	8-OH-dG [ng/d]	ELISA	healthy 10399 (1600) * obese 14879 (2720) *	10319 (2480) * 12639 (2240) *	--	--	--	--	[61]
Samjoo 2013		isoprostane [ng/day]	ELISA	healthy 1087 (104) * obese 1479 (272) *	935 (88) * 959 (136) *	--	--	--	--	
Santilli 2013	24 h	isoprostane [pg/mg crea]	RIA	325 (287–508) *	218 159–335) *	--	--	--	--	[62]
Schmitz 2008	24 h	isoprostane [pmol/mg crea]	GC-MS	78.79 (52.13) ^§^	52.19 (19.17) ^§^	--	--	--	--	[63]
Sumida 1997	24 h	8-OH-dG [nmol/mmol crea]	HPLC	treadmill exhaustion 1.67 (0.18) * bycycle exhaustion 1.93 (0.09) * running for 20 km 1.66 (0.16) *	1.61 (0.17) * 1.89 (0.18) * 1.49 (0.16) *	--	--	--	--	[64]
Vempati 2009	spot	isoprostane [pg/mg crea]	ELISA	455.4 (991) ^†^	26.9 (210) ^†^	--	--	--	--	[65]
Vezzoli 2014	spot	8-OH-dG [ng/mg crea]	ELISA	moderate intensity 5.50 (0.66) ^§^ high intensity 4.52 (0.50) ^§^	4.16 (0.40) ^§^ 3.18 (0.34) ^§^	--	--	--	--	[66]
Vezzoli 2016	spot	isoprostane [ng/mg crea]	ELISA	50 km running 0.42 (0.13) ^§^ 100 km running 0.40 (0.13) ^§^	0.60 (0.14) ^§^ 0.94 (0.13) ^§^	--	--	--	--	[67]
Vezzoli 2016		8-OH-dG [ng/mg crea]	ELISA	50 km running 4.38 (1.16) ^§^ 100 km running 4.50 (0.94) ^§^	7.48 (1.16) ^§^ 11.61 (1.18) ^§^	--	--	--	--	
Viana-Gomes 2018	spot	--	--	--	--	TBARS [umol/L]	Colorimetric	2.1 (0.3) *	3.1 (0.4) *	[68]
Viana-Gomes 2018		--	--	--	--	TAC [umol/L]	Colorimetric	41.8 (2.3) *	44.2 (2.8) *	
Viana-Gomes 2018		--	--	--	--	UA [IU/dL]	Colorimetric	2.5 (0.3) *	3.1 (0.4) *	
Viguie 1993	24 h	8-OH-dG [pmol/kg/day]	HPLC	405.3 (44.8) *	306.9 (54) *	--	--	--	--	[69]

## Data Availability

The data presented in this study are available in original articles that were included in the systematic review and meta-analysis.

## Supplementary Materials

The following are available online at https://www.mdpi.com/article/10.3390/antiox10122008/s1, Figure S1: Publication bias test for meta-analysis focusing on the effect of physical activity on urinary 8-oxo-dG or 8-OH-dG, evaluated by Funnel plot and Hedges’ g (not significant), both indicating absence of publication bias; Figure S2: Publication bias test for meta-analysis focusing on the effect of physical activity on urinary isoprostanes, evaluated by Funnel plot and Hedges’ g (not significant), both indicating absence of publication bias.

## Appendix A

Search strategy used to interrogate electronic databases.

**PubMed**

#1   "Oxidative Stress"[Mesh]

#2   "Antioxidants"[Mesh]

#3   oxidative[tiab] OR oxidation[tiab] OR oxidant[tiab] OR anti-oxidant[tiab] OR antioxidant[tiab] OR antioxidative[tiab] OR anti-oxidative[tiab]

#4   #1 OR #2 OR #3

#5   "Malondialdehyde"[Mesh]

#6   malondialdehyde[tiab] OR malonylaldehyde[tiab] OR malonaldehyde[tiab] OR malonyldialdehyde[tiab] OR MDA[tiab]

#7   #5 OR #6

#8   "8-Hydroxy-2'-Deoxyguanosine"[Mesh]

#9   8-hydroxy-2'-deoxyguanosine[tiab] OR 8-hydroxy-deoxyguanosine[tiab] OR 8-hydroxydeoxyguanosine[tiab] OR 8-hydroxyguanine[tiab] OR 8-hydroxy-guanine[tiab] OR 8-Oxo-2'-Deoxyguanosine[tiab] OR 8-Oxo-Deoxyguanosine[tiab] OR 8-oxo-dGuo[tiab] OR 8-Ohdg[tiab] OR 8OHdG[tiab] OR 8-OH-dG[tiab] OR 8-ohg[tiab] OR 8-hydroxy-g[tiab] OR 8-hydroxy-dg[tiab] OR 8-oxodG[tiab] OR 8-oxodGuo[tiab] OR 8-oxo-dG[tiab] OR 8-OH-2dG*[tiab] OR 8-isoprostane*[tiab]

#10   #8 OR #9

#11   "F2-Isoprostanes"[Mesh]

#12   IsoP[tiab] OR F2-isoprostane*[tiab]

#13   #11 OR #12

#14   "Dinoprost"[Mesh]

#15   dinoprost[tiab] OR 15-f2t-isop[tiab] OR 8-iso-PGF2a[tiab] OR 8-isoprostaglandin-f2[tiab] OR 8-iso-prostaglandin-f2[tiab] OR 8-iso-PGF2a[tiab] OR 8-epi-prostaglandin-F2alpha[tiab] OR 8-epi-prostaglandin-f2alpha[tiab] OR 8-epiprostaglandin-f2alpha[tiab] OR 8-epi-PGF2alpha[tiab]

#16   #14 OR #15

#17   "Allantoin"[Mesh]

#18   allantoin*[tiab] OR 2,5-dioxo-4-imidazolidinyl*[tiab] OR glyoxyldiureide*[tiab] OR 5-ureidohydantoin*[tiab]

#19   #17 OR #18

#20   total-antioxidant-capacity[tiab] OR total-anti-oxidant-capacity[tiab] OR total-antioxidant-power[tiab] OR total-anti-oxidant-power[tiab]

#21   "Thiobarbituric Acid Reactive Substances"[Mesh]

#22   TBARS[tiab] OR thiobarbituric-acid-reactive-substance*[tiab]

#23   #21 OR #22

#24   "Glutathione"[Mesh]

#25   "Glutathione Peroxidase"[Mesh]

#26   glutathion*[tiab] OR GSH[tiab] OR GSSH[tiab] OR GSH/GSSG[tiab] OR GPX[tiab]

#27   #24 OR #25 OR #26

#28   "Uric Acid"[Mesh]

#29   uric-acid[tiab] OR UA[tiab]

#30   #28 OR #29

#31   "Superoxide Dismutase"[Mesh]

#32   dismutase*[tiab] OR SOD[tiab]

#33   #31 OR #32

#34   "Lipid Peroxides"[Mesh]

#35   lipid-peroxid*[tiab] OR hydroperoxid*[tiab] OR lipoperoxid*[tiab]

#36   #34 OR #35

#37   "Advanced Oxidation Protein Products"[Mesh]

#38   AOPPs[tiab]

#39   #37 OR #38

#40   "Glycation End Products, Advanced"[Mesh]

#41   glycation-endproduct*[tiab] OR glycation-end-product*[tiab] OR maillard*[tiab]

#42   #40 OR #41

#43   "dityrosine"[Supplementary Concept]

#44   dityrosin*[tiab] OR bityrosin*[tiab]

#45   #43 OR #44

#46   "4-oxo-2-nonenal"[Supplementary Concept]

#47   4-oxo-2-nonenal*[tiab] OR 4-oxonon-2-enal*[tiab] OR 4-ONE[tiab]

#48   #46 OR #47

#49   "Acrolein"[Mesh]

#50   acrolein*[tiab] OR acraldehyd*[tiab] OR acrylic-aldehyd*[tiab] OR 2-propenal*[tiab]

#51   #49 OR #50

#52   "4-hydroxy-2-nonenal"[Supplementary Concept]

#53   4-hydroxy-2-nonenal[tiab] OR 4-hydroxynonen-2-al[tiab] OR 4-HNE[tiab] OR 4-hydroxynonenal[tiab]

#54   #52 OR #53

#55   #4 OR #7 OR #10 OR #13 OR #16 OR #19 OR #20 OR #23 OR #27 OR #30 OR #33 OR #36 OR #39 OR #42 OR #45 OR #48 OR #51 OR #54

#56   "Exercise"[Mesh]

#57   "Physical Exertion"[Mesh]

#58   "Physical Functional Performance"[Mesh]

#59   "Sports"[Mesh]

#60   "Athletes"[Mesh]

#61   "Leisure Activities"[Mesh]

#62   physical[tiab] AND (activit*[tiab] OR exertion[tiab])

#63   exercise*[tiab] OR training[tiab] OR fitness[tiab] OR endurance[tiab]

#64   sport*[tiab] OR gymn*[tiab] OR running[tiab] OR runner*[tiab] OR athlet*[tiab] OR marathon*[tiab] OR jogg*[tiab] OR swimm*[tiab] OR walking[tiab] OR walker*[tiab] OR leisure*[tiab] OR treadmill*[tiab] OR bicycl*[tiab] OR volley*[tiab] OR soccer*[tiab] OR football*[tiab]

#65   #56 OR #57 OR #58 OR #59 OR #60 OR #61 OR #62 OR #63 OR #64

#66   urine[subheading]

#67   "Urine"[Mesh]

#68   "Urinalysis"[Mesh]

#69   urine[tiab] OR urines[tiab] OR urinary[tiab] OR urinalys*[tiab]

#70   "Saliva"[Mesh]

#71   saliva*[tiab] OR oral-fluid*[tiab]

#72   non-invasive*[tiab] OR non-intrusive*[tiab] OR noninvasive*[tiab] OR nonintrusive*[tiab]

#73   micro-invasive*[tiab] OR microinvasive*[tiab]

#74   #66 OR #67 OR #68 OR #69 OR #70 OR #71 OR #72 OR #73

#75   #55 AND #65 AND #74

**Embase (embase.com platform)**

#1   'oxidative stress'/exp

#2   'antioxidant'/exp

#3   oxidative:ti,ab,kw OR oxidation:ti,ab,kw OR oxidant:ti,ab,kw OR anti-oxidant:ti,ab,kw OR antioxidant:ti,ab,kw OR antioxidative:ti,ab,kw OR anti-oxidative:ti,ab,kw

#4   #1 OR #2 OR #3

#5   'malonaldehyde'/exp

#6   malondialdehyde:ti,ab,kw OR malonylaldehyde:ti,ab,kw OR malonaldehyde:ti,ab,kw OR malonyldialdehyde:ti,ab,kw OR MDA:ti,ab,kw

#7   #5 OR #6

#8   '8 hydroxydeoxyguanosine'/exp

#9   8-hydroxy-2-deoxyguanosine:ti,ab,kw OR 8-hydroxy-deoxyguanosine:ti,ab,kw OR 8-hydroxydeoxyguanosine:ti,ab,kw OR 8-hydroxyguanine:ti,ab,kw OR 8-hydroxy-guanine:ti,ab,kw OR 8-Oxo-2-Deoxyguanosine:ti,ab,kw OR 8-Oxo-Deoxyguanosine:ti,ab,kw OR 8-oxo-dGuo:ti,ab,kw OR 8-Ohdg:ti,ab,kw OR 8OHdG:ti,ab,kw OR 8-OH-dG:ti,ab,kw OR 8-ohg:ti,ab,kw OR 8-hydroxy-g:ti,ab,kw OR 8-hydroxy-dg:ti,ab,kw OR 8-oxodG:ti,ab,kw OR 8-oxodGuo:ti,ab,kw OR 8-oxo-dG:ti,ab,kw OR 8-OH-2dG*:ti,ab,kw OR 8-isoprostane*:ti,ab,kw

#10   #8 OR #9

#11   'isoprostane derivative'/exp

#12   IsoP:ti,ab,kw OR F2-isoprostane*:ti,ab,kw

#13   #11 OR #12

#14   'prostaglandin E2'/exp

#15   dinoprost:ti,ab,kw OR 15-f2t-isop:ti,ab,kw OR 8-iso-PGF2a:ti,ab,kw OR 8-isoprostaglandin-f2:ti,ab,kw OR 8-iso-prostaglandin-f2:ti,ab,kw OR 8-iso-PGF2a:ti,ab,kw OR 8-epi-prostaglandin-F2alpha:ti,ab,kw OR 8-epi-prostaglandin-f2alpha:ti,ab,kw OR 8-epiprostaglandin-f2alpha:ti,ab,kw OR 8-epi-PGF2alpha:ti,ab,kw

#16   #14 OR #15

#17   'allantoin'/exp

#18   allantoin*:ti,ab,kw OR dioxo-4-imidazolidinyl*:ti,ab,kw OR glyoxyldiureide*:ti,ab,kw OR 5-ureidohydantoin*:ti,ab,kw

#19   #17 OR #18

#20   total-antioxidant-capacity:ti,ab,kw OR total-anti-oxidant-capacity:ti,ab,kw OR total-antioxidant-power:ti,ab,kw OR total-anti-oxidant-power:ti,ab,kw

#21   'thiobarbituric acid reactive substance'/exp

#22   TBARS:ti,ab,kw OR thiobarbituric-acid-reactive-substance*:ti,ab,kw

#23   #21 OR #22

#24   'glutathione'/exp

#25   'glutathione peroxidase'/exp

#26   glutathion*:ti,ab,kw OR GSH:ti,ab,kw OR GSSH:ti,ab,kw OR GSSG:ti,ab,kw OR GPX:ti,ab,kw

#27   #24 OR #25 OR #26

#28   'uric acid'/exp

#29   uric-acid:ti,ab,kw OR UA:ti,ab,kw

#30   #28 OR #29

#31   'superoxide dismutase'/exp

#32   dismutase*:ti,ab,kw OR SOD:ti,ab,kw

#33   #31 OR #32

#34   'lipid peroxide'/exp

#35   lipid-peroxid*:ti,ab,kw OR hydroperoxid*:ti,ab,kw OR lipoperoxid*:ti,ab,kw

#36   #34 OR #35

#37   'advanced oxidation protein product'/exp

#38   AOPPs:ti,ab,kw

#39   #37 OR #38

#40   'advanced glycation end product'/exp

#41   glycation-endproduct*:ti,ab,kw OR glycation-end-product*:ti,ab,kw OR maillard*:ti,ab,kw

#42   #40 OR #41

#43   dityrosin*:ti,ab,kw OR bityrosin*:ti,ab,kw

#44   4-oxo-2-nonenal*:ti,ab,kw OR 4-oxonon-2-enal*:ti,ab,kw OR 4-ONE:ti,ab,kw

#45   'acrolein'/exp

#46   acrolein*:ti,ab,kw OR acraldehyd*:ti,ab,kw OR acrylic-aldehyd*:ti,ab,kw OR 2-propenal*:ti,ab,kw

#47   #45 OR #46

#48   '4 hydroxynonenal'/exp

#49   4-hydroxy-2-nonenal:ti,ab,kw OR 4-hydroxynonen-2-al:ti,ab,kw OR 4-HNE:ti,ab,kw OR 4-hydroxynonenal:ti,ab,kw

#50   #48 OR #49

#51   #4 OR #7 OR #10 OR #13 OR #16 OR #19 OR #20 OR #23 OR #27 OR #30 OR #33 OR #36 OR #39 OR #42 OR #43 OR #44 OR #47 OR #50

#52   'physical activity, capacity and performance'/exp

#53   'sport'/exp

#54   'athlete'/exp

#55   'recreation'/exp

#56   physical:ti,ab,kw AND (activit*:ti,ab,kw OR exertion:ti,ab,kw)

#57   exercise*:ti,ab,kw OR training:ti,ab,kw OR fitness:ti,ab,kw OR endurance:ti,ab,kw

#58   sport*:ti,ab,kw OR gymn*:ti,ab,kw OR running:ti,ab,kw OR runner*:ti,ab,kw OR athlet*:ti,ab,kw OR marathon*:ti,ab,kw OR jogg*:ti,ab,kw OR swimm*:ti,ab,kw OR walking:ti,ab,kw OR walker*:ti,ab,kw OR leisure*:ti,ab,kw OR treadmill*:ti,ab,kw OR bicycl*:ti,ab,kw OR volley*:ti,ab,kw OR soccer*:ti,ab,kw OR football*:ti,ab,kw

#59   #52 OR #53 OR #54 OR #55 OR #56 OR #57 OR #58

#60   'urine'/exp

#61   'urinalysis'/exp

#62   urine:ti,ab,kw OR urines:ti,ab,kw OR urinary:ti,ab,kw OR urinalys*:ti,ab,kw

#63   'saliva'/exp

#64   saliva*:ti,ab,kw OR oral-fluid*:ti,ab,kw

#65   non-invasive*:ti,ab,kw OR non-intrusive*:ti,ab,kw OR noninvasive*:ti,ab,kw OR nonintrusive*:ti,ab,kw

#66   micro-invasive*:ti,ab,kw OR microinvasive*:ti,ab,kw

#67   #60 OR #61 OR #62 OR #63 OR #64 OR #65 OR #66

#68   #51 AND #59 AND #67

**Cochrane Central Register of Controlled Trials (CENTRAL)**

#1   MeSH descriptor: [Oxidative Stress] explode all trees

#2   MeSH descriptor: [Antioxidants] explode all trees

#3   (oxidative OR oxidation OR oxidant OR anti-oxidant OR antioxidant OR antioxidative OR anti- oxidative):ti,ab,kw

#4   MeSH descriptor: [Malondialdehyde] explode all trees

#5   (malondialdehyde OR malonylaldehyde OR malonaldehyde OR malonyldialdehyde OR MDA):ti,ab,kw

#6   MeSH descriptor: [8-Hydroxy-2'-Deoxyguanosine] explode all trees

#7   ("8-hydroxy-2'-deoxyguanosine" OR "8-hydroxy-deoxyguanosine" OR "8-hydroxydeoxyguanosine" OR "8-hydroxyguanine" OR "8-hydroxy-guanine" OR "8-Oxo-2'-Deoxyguanosine"):ti,ab,kw

#8   ("8-Oxo-Deoxyguanosine" OR "8-oxo-dGuo" OR "8-Ohdg" OR 8OHdG OR "8-OH-dG" OR "8-ohg" OR "8-hydroxy-g" OR "8-hydroxy-dg" OR "8-oxodG" OR "8-oxodGuo" OR "8-oxo-dG" OR "8-OH-2dG" OR "8-isoprostane"):ti,ab,kw

#9   MeSH descriptor: [F2-Isoprostanes] explode all trees

#10   (IsoP OR "F2-isoprostane"):ti,ab,kw

#11   MeSH descriptor: [Dinoprost] explode all trees

#12   (dinoprost OR "15-f2t-isop" OR "8-iso-PGF2a" OR "8-isoprostaglandin-f2" OR "8-iso-prostaglandin- f2" OR "8-iso-PGF2a" OR "8-epi-prostaglandin-F2alpha" OR "8-epi-prostaglandin-f2alpha" OR "8- epiprostaglandin-f2alpha" OR "8-epi-PGF2alpha"):ti,ab,kw

#13   MeSH descriptor: [Allantoin] explode all trees

#14   (allantoin* OR "2,5-dioxo-4-imidazolidinyl" OR glyoxyldiureide OR "5-ureidohydantoin" OR "total antioxidant capacity" OR "total anti-oxidant capacity" OR "total antioxidant power" OR "total anti- oxidant power"):ti,ab,kw

#15   MeSH descriptor: [Thiobarbituric Acid Reactive Substances] explode all trees

#16   (TBARS OR "thiobarbituric acid reactive substances"):ti,ab,kw

#17   MeSH descriptor: [Glutathione] explode all trees

#18   (glutathion* OR GSH OR GSSH OR "GSH/GSSG" OR GPX):ti,ab,kw

#19   MeSH descriptor: [Uric Acid] explode all trees

#20   ("uric acid" OR UA):ti,ab,kw

#21   MeSH descriptor: [Superoxide Dismutase] explode all trees

#22   (dismutase* OR SOD):ti,ab,kw

#23   MeSH descriptor: [Lipid Peroxidation] explode all trees

#24   ("lipid peroxidation" OR hydroperoxid* OR lipoperoxid*):ti,ab,kw

#25   MeSH descriptor: [Advanced Oxidation Protein Products] explode all trees

#26   (AOPPs):ti,ab,kw

#27   MeSH descriptor: [Glycation End Products, Advanced] explode all trees

#28   ("glycation endproducts" OR "glycation end-products" OR maillard* OR dityrosin* OR bityrosin* OR "4-oxo-2-nonenal" OR "4-oxonon-2-enal" OR "4-ONE"):ti,ab,kw

#29   MeSH descriptor: [Acrolein] explode all trees

#30   (acrolein* OR acraldehyde* OR "acrylic aldehyde" OR "2-propenal" OR "4-hydroxy-2-nonenal" OR "4-hydroxynonen-2-al" OR "4-HNE" OR "4-hydroxynonenal"):ti,ab,kw

#31   #1 OR #2 OR #3 OR #4 OR #5 OR #6 OR #7 OR #8 OR #9 OR #10 OR #11 OR #12 OR #13 OR #14 OR #15 OR #16 OR #17 OR #18 OR #19 OR #20 OR #21 OR #22 OR #23 OR #24 OR #25 OR #26 OR #27 OR #28 OR #29 OR #30

#32   MeSH descriptor: [Exercise] explode all trees

#33   MeSH descriptor: [Physical Exertion] explode all trees

#34   MeSH descriptor: [Physical Functional Performance] explode all trees

#35   MeSH descriptor: [Sports] explode all trees

#36   MeSH descriptor: [Athletes] explode all trees

#37   MeSH descriptor: [Leisure Activities] explode all trees

#38   ((physical AND (activit* OR exertion))):ti,ab,kw

#39   (exercise* OR training OR fitness OR endurance OR sport* OR gymn* OR running OR runner* OR athlet* OR marathon* OR jogg* OR swimm* OR walking OR walker* OR leisure* OR treadmill* OR bicycl* OR volley* OR soccer* OR football*):ti,ab,kw

#40   #32 OR #33 OR #34 OR #35 OR #36 OR #37 OR #38 OR #39

#41   MeSH descriptor: [Urine] explode all trees

#42   MeSH descriptor: [Urinalysis] explode all trees

#43   (urine OR urines OR urinary OR urinalys*):ti,ab,kw

#44   MeSH descriptor: [Saliva] explode all trees

#45   (saliva* OR "oral fluid" OR "oral fluids" OR non-invasive* OR non-intrusive* OR noninvasive* OR nonintrusive* OR micro-invasive* OR microinvasive*):ti,ab,kw

#46   #41 OR #42 OR #43 OR #44 OR #45

## References

[1] Kruk J. (2007). Physical activity in the prevention of the most frequent chronic diseases: An analysis of the recent evidence. Asian Pac. J. Cancer Prev..

[2] Pizzino G., Irrera N., Cucinotta M., Pallio G., Mannino F., Arcoraci V., Squadrito F., Altavilla D., Bitto A. (2017). Oxidative Stress: Harms and Benefits for Human Health. Oxid. Med. Cell. Longev..

[3] Roy J., Galano J., Durand T., Le Guennec J., Lee J.C. (2017). Physiological role of reactive oxygen species as promoters of natural defenses. FASEB J..

[4] Kruk J., Aboul-Enein H.Y., Kładna A., Bowser J.E. (2019). Oxidative stress in biological systems and its relation with pathophysiological functions: The effect of physical activity on cellular redox homeostasis. Free Radic. Res..

[5] Dillard C.J., Litov R.E., Savin W.M., Dumelin E.E., Tappel A.L. (1978). Effects of exercise, vitamin E, and ozone on pulmonary function and lipid peroxidation. J. Appl. Physiol..

[6] Davies K.J.A., Quintanilha A.T., Brooks G.A., Packer L. (1982). Free radicals and tissue damage produced by exercise. Biochem. Biophys. Res. Commun..

[7] Reid M.B., Khawli F.A., Moody M.R. (1993). Reactive oxygen in skeletal muscle. III. Contractility of unfatigued muscle. J. Appl. Physiol..

[8] Hammeren J., Powers S., Lawler J., Criswell D., Martin D., Lowenthal D., Pollock M. (1992). Exercise Training-Induced Alterations in Skeletal Muscle Oxidative and Antioxidant Enzyme Activity in Senescent Rats. Int. J. Sports Med..

[9] Palmer R.M.J., Ferrige A.G., Moncada S. (1987). Nitric oxide release accounts for the biological activity of endothelium-derived relaxing factor. Nat. Cell Biol..

[10] Balon T.W., Nadler J.L. (1994). Nitric oxide release is present from incubated skeletal muscle preparations. J. Appl. Physiol..

[11] Radak Z., Chung H.Y., Goto S. (2005). Exercise and hormesis: Oxidative stress-related adaptation for successful aging. Biogerontology.

[12] Mattson M.P. (2008). Hormesis defined. Ageing Res. Rev..

[13] Fisher-Wellman K., Bloomer R.J. (2009). Acute exercise and oxidative stress: A 30 year history. Dyn. Med..

[14] Li G., He H. (2009). Hormesis, allostatic buffering capacity and physiological mechanism of physical activity: A new theoretic framework. Med. Hypotheses.

[15] Powers S.K., Radak Z., Ji L.L. (2016). Exercise-induced oxidative stress: Past, present and future. J. Physiol..

[16] Webb R., Hughes M.G., Thomas A.W., Morris K. (2017). The Ability of Exercise-Associated Oxidative Stress to Trigger Redox-Sensitive Signalling Responses. Antioxidants.

[17] Goto C., Higashi Y., Kimura M., Noma K., Hara K., Nakagawa K., Kawamura M., Chayama K., Yoshizumi M., Nara I. (2003). Effect of Different Intensities of Exercise on Endothelium-Dependent Vasodilation in Humans: Role of Endothelium-Dependent Nitric Oxide and Oxidative Stress. Circulation.

[18] Il’Yasova D., Scarbrough P., Spasojevic I. (2012). Urinary biomarkers of oxidative status. Clin. Chim. Acta.

[19] Thirupathi A., Pinho R.A., Ugbolue U.C., He Y., Meng Y., Gu Y. (2021). Effect of Running Exercise on Oxidative Stress Biomarkers: A Systematic Review. Front. Physiol..

[20] Page M.J., McKenzie J.E., Bossuyt P.M., Boutron I., Hoffmann T.C., Mulrow C.D., Shamseer L., Tetzlaff J.M., Akl E.A., Brennan S.E. (2021). The PRISMA 2020 statement: An updated guideline for reporting systematic reviews. BMJ.

[21] (2014). National Institutes of Health, Quality Assessment Tool. https://www.nhlbi.nih.gov/health-pro/guidelines/in-develop/cardiovascular-risk-reduction/tools/cohort.

[22] Tufanaru C., Munn Z., Aromataris E., Campbell J., Aromataris E., Munn Z. (2020). Systematic Reviews of Effectiveness.

[23] Wan X., Wang W., Liu J., Tong T. (2014). Estimating the sample mean and standard deviation from the sample size, median, range and/or interquartile range. BMC Med. Res. Methodol..

[24] Rosenthal R. (1991). Applied social research methods series. Survey Research.

[25] Olkin I., Dahabreh I.J., Trikalinos T.A. (2012). GOSH—A graphical display of study heterogeneity. Res. Synth. Methods.

[26] R Core Team (2020). R: A Language and Environment for Statistical Computing.

[27] Allgayer H., Owen R.W., Nair J., Spiegelhalder B., Streit J., Reichel C., Bartsch H. (2008). Short-term moderate exercise programs reduce oxidative DNA damage as determined by high-performance liquid chromatography-electrospray ionization-mass spectrometry in patients with colorectal carcinoma following primary treatment. Scand. J. Gastroenterol..

[28] Almar M., Villa J.G., Cuevas M.J., Rodríguez-Marroyo J.A., Avila C., Gonzalez-Gallego J. (2002). Urinary levels of 8-hydroxydeoxyguanosine as a marker of oxidative damage in road cycling. Free Radic. Res..

[29] Arazi H., Taati B., Sajedi F.R., Suzuki K. (2019). Salivary Antioxidants Status Following Progressive Aerobic Exercise: What Are the Differences between Waterpipe Smokers and Non-Smokers?. Antioxidants.

[30] Babaei P., Damirchi A., Tehrani B.S., Nazari Y., Sariri R., Hoseini R. (2016). Effect of exercise training on saliva brain derived neurotrophic factor, catalase and vitamin c. Med. J. Islam. Repub. Iran.

[31] Chielle E.O., Granella L.W., Maziero J.S., Vidigal T.M.A., Mallmann B.L.K., Karal J. (2019). Evolution of potential biomarkers of acute muscle injury after physical exercise. Braz. J. Pharm. Sci..

[32] Córdova A., Sureda A., Albina M.L., Linares V., Bellés M., Sánchez D.J. (2015). Oxidative Stress Markers After a Race in Professional Cyclists. Int. J. Sport Nutr. Exerc. Metab..

[33] Damirchi A., Kiani M., Jafarian V., Sariri R. (2010). Response of salivary peroxidase to exercise intensity. Graefe’s Arch. Clin. Exp. Ophthalmol..

[34] Deminice R., Sicchieri T., Payão P.O., Jordão A.A. (2010). Blood and Salivary Oxidative Stress Biomarkers Following an Acute Session of Resistance Exercise in Humans. Int. J. Sports Med..

[35] Devries M.C., Hamadeh M., Glover A.W., Raha S., Samjoo I.A., Tarnopolsky M.A. (2008). Endurance training without weight loss lowers systemic, but not muscle, oxidative stress with no effect on inflammation in lean and obese women. Free Radic. Biol. Med..

[36] Gargallo P., Colado J.C., Juesas A., Hernando-Espinilla A., Capell N.E., Monzó-Beltran L., García-Pérez P., Cauli O., Sáez G. (2018). The Effect of Moderate- Versus High-Intensity Resistance Training on Systemic Redox State and DNA Damage in Healthy Older Women. Biol. Res. Nurs..

[37] Hartmann A., Pfuhler S., Dennog C., Germadnik D., Pilger A., Speit G. (1998). Exercise-Induced DNA Effects in Human Leukocytes Are Not Accompanied by Increased Formation of 8-Hydroxy-2′-Deoxyguanosine or Induction of Micronuclei. Free Radic. Biol. Med..

[38] Hofer T., Fontana L., Anton S.D., Weiss E.P., Villareal D., Malayappan B., Leeuwenburgh C. (2008). Long-Term Effects of Caloric Restriction or Exercise on DNA and RNA Oxidation Levels in White Blood Cells and Urine in Humans. Rejuvenation Res..

[39] Jackson R., Ramos C., Gupta C., Gomez-Marin O. (2010). Exercise decreases plasma antioxidant capacity and increases urinary isoprostanes of IPF patients. Respir. Med..

[40] Karstoft K., Clark M.A., Jakobsen I., Müller I.A., Pedersen B.K., Solomon T., Ried-Larsen M. (2017). The effects of 2 weeks of interval vs continuous walking training on glycaemic control and whole-body oxidative stress in individuals with type 2 diabetes: A controlled, randomised, crossover trial. Diabetologia.

[41] Lessiani G., Santilli F., Boccatonda A., Iodice P., Liani R., Tripaldi R., Saggini R., Davì G. (2016). Arterial stiffness and sedentary lifestyle: Role of oxidative stress. Vasc. Pharmacol..

[42] Margaritelis N., Kyparos A., Paschalis V., Theodorou A., Panayiotou G., Zafeiridis A., Dipla K., Nikolaidis M., Vrabas I. (2014). Reductive stress after exercise: The issue of redox individuality. Redox Biol..

[43] Margonis K., Fatouros I.G., Jamurtas T., Nikolaidis M.G., Douroudos I., Chatzinikolaou A., Mitrakou A., Mastorakos G., Papassotiriou I., Taxildaris K. (2007). Oxidative stress biomarkers responses to physical overtraining: Implications for diagnosis. Free Radic. Biol. Med..

[44] Medina S., Domínguez-Perles R., Cejuela-Anta R., Villaño D., Martínez-Sanz J.M., Gil P., García-Viguera C., Ferreres F., Gil J.I., Gil-Izquierdo A. (2012). Assessment of oxidative stress markers and prostaglandins after chronic training of triathletes. Prostaglandins Other Lipid Mediat..

[45] Mendoza-Núñez V.M., Hernández-Monjaraz B., Santiago-Osorio E., Betancourt-Rule J.M., Ruiz-Ramos M. (2014). Tai Chi Exercise Increases SOD Activity and Total Antioxidant Status in Saliva and Is Linked to an Improvement of Periodontal Disease in the Elderly. Oxidative Med. Cell. Longev..

[46] Mercken E.M., Hageman G.J., Schols A.M.W.J., Akkermans M.A., Bast A., Wouters E.F.M. (2005). Rehabilitation Decreases Exercise-induced Oxidative Stress in Chronic Obstructive Pulmonary Disease. Am. J. Respir. Crit. Care Med..

[47] Mikami T., Kita K., Tomita S., Qu G.-J., Tasaki Y., Ito A. (2000). Is allantoin in serum and urine a useful indicator of exercise-induced oxidative stress in humans?. Free Radic. Res..

[48] Nemoto K., Oh-Ishi S., Itoh M., Saito T., Ichiwata T. (2014). Urinary 8-Hydroxydeoxyguanosine Is a Potential Indicator for Estimating Pulmonary Rehabilitation-Induced Oxidative Stress in COPD Patients. Tohoku J. Exp. Med..

[49] Nikolaidis M.G., Kyparos A., Dipla K., Zafeiridis A., Sambanis M., Grivas G., Paschalis V., Theodorou A.A., Papadopoulos S., Spanou C. (2012). Exercise as a model to study redox homeostasis in blood: The effect of protocol and sampling point. Biomarkers.

[50] Nikolaidis M.G., Kyparos A., Spanou C., Paschalis V., Theodorou A.A., Panayiotou G., Grivas G., Zafeiridis A., Dipla K., Vrabas I.S. (2013). Aging is not a barrier to muscle and redox adaptations: Applying the repeated eccentric exercise model. Exp. Gerontol..

[51] Nojima H., Watanabe H., Yamane K., Kitahara Y., Sekikawa K., Yamamoto H., Yokoyama A., Inamizu T., Asahara T., Kohno N. (2008). Effect of aerobic exercise training on oxidative stress in patients with type 2 diabetes mellitus. Metabolism.

[52] Orhan H., van Holland B., Krab B., Moeken J., Vermeulen N.P., Hollander P., Meerman J.H. (2004). Evaluation of a Multi-parameter Biomarker Set for Oxidative Damage in Man: Increased Urinary Excretion of Lipid, Protein and DNA Oxidation Products after One Hour of Exercise. Free Radic. Res..

[53] Parise G., Brose A.N., Tarnopolsky M.A. (2005). Resistance exercise training decreases oxidative damage to DNA and increases cytochrome oxidase activity in older adults. Exp. Gerontol..

[54] Poulsen S.L.H.E. (1996). Extreme exercise and oxidative DNA modification. J. Sports Sci..

[55] Radák Z., Pucsuk J., Boros S., Josfai L., Taylor A. (2000). Changes in urine 8-hydroxydeoxyguanosine levels of super-marathon runners during a four-day race period. Life Sci..

[56] Rall L.C., Roubenoff R., Meydani S.N., Han S.N., Meydani M. (2000). Urinary 8-hydroxy-2′-deoxyguanosine (8-OHdG) as a marker of oxidative stress in rheumatoid arthritis and aging: Effect of progressive resistance training. J. Nutr. Biochem..

[57] Rietjens S.J., Beelen M., Koopman R., VAN Loon L.J.C., Bast A., Haenen G.R.M.M. (2007). A Single Session of Resistance Exercise Induces Oxidative Damage in Untrained Men. Med. Sci. Sports Exerc..

[58] De Araujo V.R., Lisboa P., Boaventura G., Caramez F., Pires L., Oliveira E., Moura E., Casimiro-Lopes G. (2018). Acute high-intensity exercise test in soccer athletes affects salivary biochemical markers. Free Radic. Res..

[59] Rowlands D.S., Pearce E., Aboud A., Gillen J.B., Gibala M.J., Donato S., Waddington J.M., Green J.G., Tarnopolsky M.A. (2012). Oxidative stress, inflammation, and muscle soreness in an 894-km relay trail run. Graefe’s Arch. Clin. Exp. Ophthalmol..

[60] Samia B.A.A., Youssef G.A. (2014). Changes in Urinary 8-Hydroxydeoxyguanosine Levels During Heptathlon Race in Professional Female Athletes. J. Hum. Kinet..

[61] Samjoo I.A., Safdar A., Hamadeh M.J., Raha S., Tarnopolsky M.A. (2013). The effect of endurance exercise on both skeletal muscle and systemic oxidative stress in previously sedentary obese men. Nutr. Diabetes.

[62] Santilli F., Vazzana N., Iodice P., Lattanzio S., Liani R., Bellomo R.G., Lessiani G., Perego F., Saggini R., Davì G. (2013). Effects of high-amount–high-intensity exercise on in vivo platelet activation: Modulation by lipid peroxidation and AGE/RAGE axis. Thromb. Haemost..

[63] Schmitz K.H., Warren M., Rundle A.G., Williams N.I., Gross M.D., Kurzer M.S. (2008). Exercise Effect on Oxidative Stress Is Independent of Change in Estrogen Metabolism. Cancer Epidemiol. Biomark. Prev..

[64] Sumida S., Okamura K., Doi T., Sakurai M., Yoshioka Y., Sugawa-Katayama Y. (1997). No influence of a single bout of exercise on urinary excretion of 8-hydroxy-deoxyguanosine in humans. IUBMB Life.

[65] Vempati R., Bijlani R.L., Deepak K.K. (2009). The efficacy of a comprehensive lifestyle modification programme based on yoga in the management of bronchial asthma: A randomized controlled trial. BMC Pulm. Med..

[66] Vezzoli A., Pugliese L., Marzorati M., Serpiello F.R., La Torre A., Porcelli S. (2014). Time-Course Changes of Oxidative Stress Response to High-Intensity Discontinuous Training versus Moderate-Intensity Continuous Training in Masters Runners. PLoS ONE.

[67] Vezzoli A., Dellanoce C., Mrakic-Sposta S., Montorsi M., Moretti S., Tonini A., Pratali L., Accinni R. (2016). Oxidative Stress Assessment in Response to Ultraendurance Exercise: Thiols Redox Status and ROS Production according to Duration of a Competitive Race. Oxidative Med. Cell. Longev..

[68] Viana-Gomes D., Rosa F., Mello R., Paz G., Miranda H., Salerno V. (2018). Oxidative stress, muscle and liver cell damage in professional soccer players during a 2-game week schedule. Sci. Sports.

[69] Viguie C.A., Frei B., Shigenaga M.K., Ames B.N., Packer L., Brooks G.A. (1993). Antioxidant status and indexes of oxidative stress during consecutive days of exercise. J. Appl. Physiol..

[70] Wragg C.B., Maxwell N.S., Doust J.H. (2000). Evaluation of the reliability and validity of a soccer-specific field test of repeated sprint ability. Graefe’s Arch. Clin. Exp. Ophthalmol..

[71] Thirupathi A., Wang M., Lin J.K., Fekete G., István B., Baker J.S., Gu Y. (2021). Effect of Different Exercise Modalities on Oxidative Stress: A Systematic Review. BioMed. Res. Int..

[72] Marquez D.X., Aguiñaga S., Vásquez P.M., Conroy D.E., Erickson K.I., Hillman C., Stillman C.M., Ballard R.M., Sheppard B.B., Petruzzello S.J. (2020). A systematic review of physical activity and quality of life and well-being. Transl. Behav. Med..

[73] Viitala P., Newhouse I.J. (2004). Vitamin E supplementation, exercise and lipid peroxidation in human participants. Graefe’s Arch. Clin. Exp. Ophthalmol..

[74] Vollaard N.B.J., Shearman J., Cooper C. (2005). Exercise-Induced Oxidative Stress. Sports Med..

[75] Kawamura T., Muraoka I. (2018). Exercise-Induced Oxidative Stress and the Effects of Antioxidant Intake from a Physiological Viewpoint. Antioxidants.

[76] Silva D., Arend E., Rocha S.M., Rudnitskaya A., Delgado L., Moreira A., Carvalho M.J. (2019). The impact of exercise training on the lipid peroxidation metabolomic profile and respiratory infection risk in older adults. Eur. J. Sport Sci..

[77] Nikolaidis M.G., Kyparos A., Vrabas I.S. (2011). F2-isoprostane formation, measurement and interpretation: The role of exercise. Prog. Lipid Res..

[78] Urso M.L., Clarkson P.M. (2003). Oxidative stress, exercise, and antioxidant supplementation. Toxicology.

[79] Sacheck J.M., Blumberg J.B. (2001). Role of vitamin E and oxidative stress in exercise. Nutrition.

[80] Powers S.K., Deminice R., Ozdemir M., Yoshihara T., Bomkamp M.P., Hyatt H. (2020). Exercise-induced oxidative stress: Friend or foe?. J. Sport Health Sci..

[81] Shi M., Wang X., Yamanaka T., Ogita F., Nakatani K., Takeuchi T. (2007). Effects of anaerobic exercise and aerobic exercise on biomarkers of oxidative stress. Environ. Health Prev. Med..

[82] Bloomer R.J., Goldfarb A.H. (2004). Anaerobic Exercise and Oxidative Stress: A Review. Can. J. Appl. Physiol..

[83] Radak Z., Taylor A.W., Ohno H., Goto S. (2001). Adaptation to exercise-induced oxidative stress: From muscle to brain. Exerc. Immunol. Rev..

[84] Elejalde E., Villarán M.C., Alonso R.M. (2021). Grape polyphenols supplementation for exercise-induced oxidative stress. J. Int. Soc. Sports Nutr..

[85] Simioni C., Zauli G., Martelli A.M., Vitale M., Sacchetti G., Gonelli A., Neri L.M. (2018). Oxidative stress: Role of physical exercise and antioxidant nutraceuticals in adulthood and aging. Oncotarget.

[86] Powers S.K., Jackson M.J. (2008). Exercise-Induced Oxidative Stress: Cellular Mechanisms and Impact on Muscle Force Production. Physiol. Rev..

[87] Michailidis Y., Jamurtas A.Z., Nikolaidis M.G., Fatouros I.G., Koutedakis Y., Papassotiriou I., Kouretas D. (2007). Sampling Time is Crucial for Measurement of Aerobic Exercise-Induced Oxidative Stress. Med. Sci. Sports Exerc..

[88] Martinez-Moral M.-P., Kannan K. (2019). How stable is oxidative stress level? An observational study of intra- and inter-individual variability in urinary oxidative stress biomarkers of DNA, proteins, and lipids in healthy individuals. Environ. Int..

[89] Marrocco I., Altieri F., Peluso I. (2017). Measurement and Clinical Significance of Biomarkers of Oxidative Stress in Humans. Oxidative Med. Cell. Longev..

[90] Graille M., Wild P., Sauvain J.-J., Hemmendinger M., Canu I.G., Hopf N. (2020). Urinary 8-isoprostane as a biomarker for oxidative stress. A systematic review and meta-analysis. Toxicol. Lett..

[91] Wang J., Schipper H.M., Velly A.M., Mohit S., Gornitsky M. (2015). Salivary biomarkers of oxidative stress: A critical review. Free Radic. Biol. Med..

