# Supplementary material for: Non-Invasive Measurement of Exercise-Induced Oxidative Stress in Response to Physical Activity. A Systematic Review and Meta-Analysis
Source: Antioxidants (Basel). 2021 Dec 17;10(12):2008. doi: 10.3390/antiox10122008 (PMC8698343; doi:10.3390/antiox10122008)
Supplement: Supplementary file 1 [file antioxidants-10-02008-s001.zip › antioxidants-1492673-supplementary.pdf]

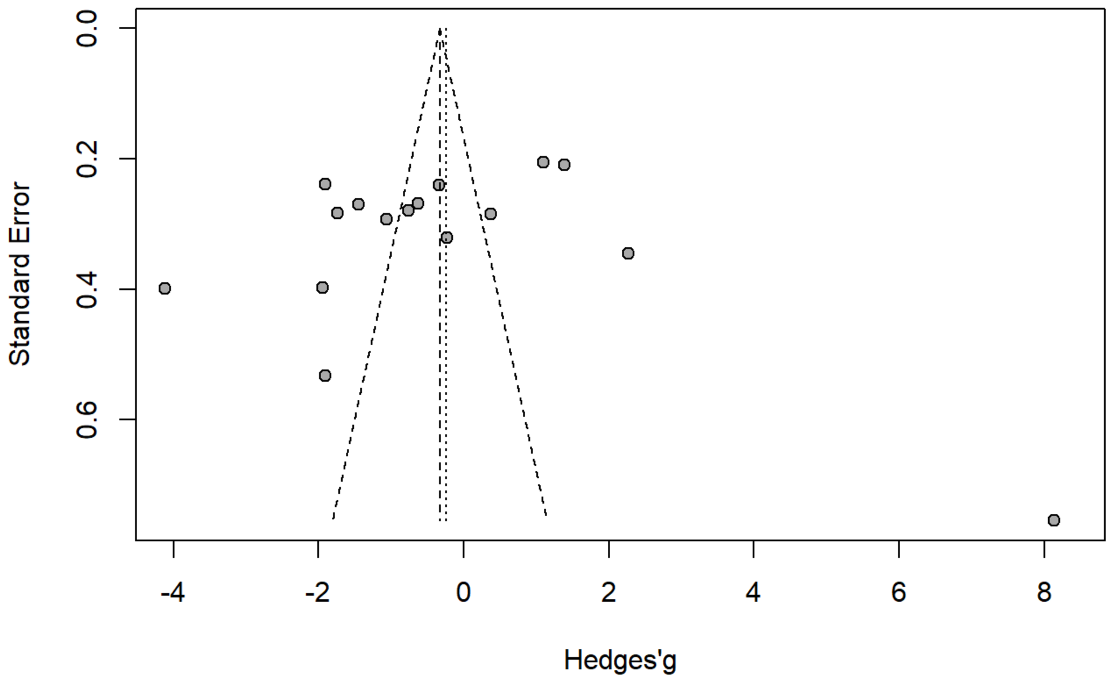

Figure S1: Publication bias test for meta-analysis focusing on the effect of physical activity on urinary 8-oxo-dG or 8-OH-dG, evaluated by Funnel plot and Hedges' g (not significant), both indi-cating absence of publication bias.

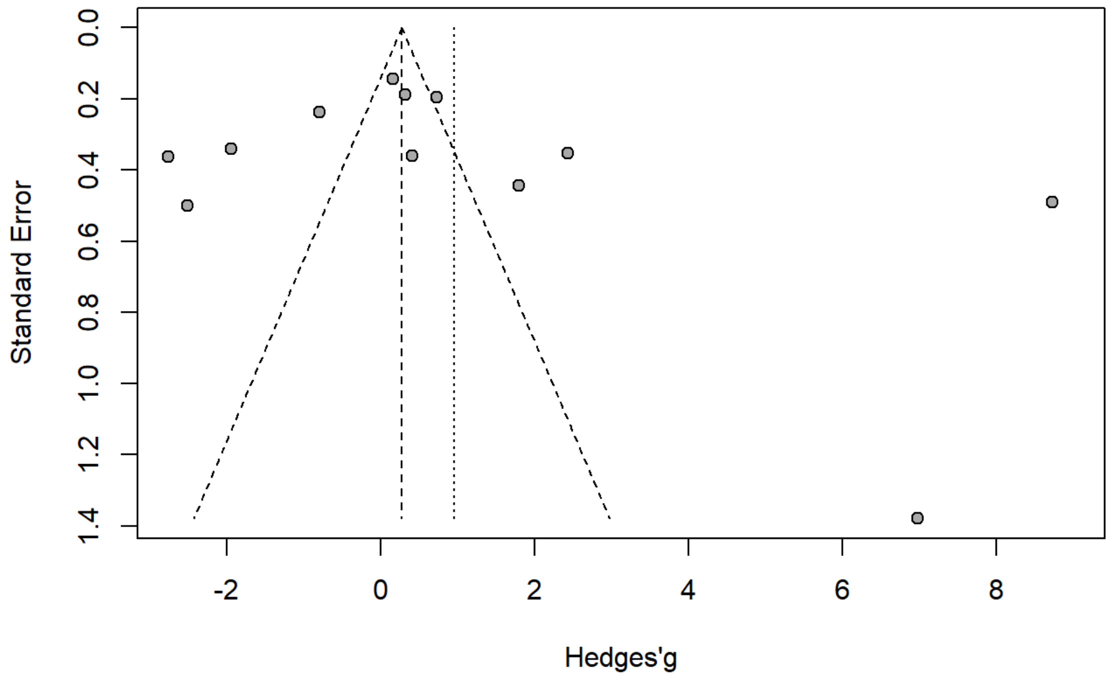

Figure S2: Publication bias test for meta-analysis focusing on the effect of physical activity on urinary isoprostanes, evaluated by Funnel plot and Hedges' g (not significant), both indicating ab-sence of publication bias.
